# *Helicobacter pylori* PqqE is a new virulence factor that cleaves junctional adhesion molecule A and disrupts gastric epithelial integrity

**DOI:** 10.1080/19490976.2021.1921928

**Published:** 2021-05-10

**Authors:** Miguel S. Marques, Ana C. Costa, Hugo Osório, Marta L. Pinto, Sandra Relvas, Mário Dinis-Ribeiro, Fátima Carneiro, Marina Leite, Ceu Figueiredo

**Affiliations:** a i3S – Instituto de Investigação e Inovação em Saúde, Universidade do Porto, Porto, Portugal; b Ipatimup – Institute of Molecular Pathology and Immunology of the University of Porto, Porto, Portugal; c Faculty of Medicine of the University of Porto, Porto, Portugal; d Department of Pathology, Centro Hospitalar Universitário S. João, Porto, Portugal; e Instituto Português de Oncologia, Porto, Portugal; f Center for Health Technology and Services Research (CINTESIS), Porto, Portugal

**Keywords:** *Helicobacter pylori* pathogenesis, bacterial proteases, bacteria-host interactions, junctional adhesion molecule A (JAM-A)/F11R, proteomics, PqqE

## Abstract

*Helicobacter pylori* infects approximately half of the world’s population and is the strongest risk factor for peptic ulcer disease and gastric cancer, representing a major global health concern. *H. pylori* persistently colonizes the gastric epithelium, where it subverts the highly organized structures that maintain epithelial integrity. Here, a unique strategy used by *H. pylori* to disrupt the gastric epithelial junctional adhesion molecule-A (JAM-A) is disclosed, using various experimental models that include gastric cell lines, primary human gastric cells, and biopsy specimens of infected and non-infected individuals. *H. pylori* preferentially cleaves the cytoplasmic domain of JAM-A at Alanine 285. Cells stably transfected with full-length JAM-A or JAM-A lacking the cleaved sequence are used in a range of functional assays, which demonstrate that the *H. pylori* cleaved region is critical to the maintenance of the epithelial barrier and of cell-cell adhesion. Notably, by combining chromatography techniques and mass spectrometry, PqqE (HP1012) is purified and identified as the *H. pylori* virulence factor that cleaves JAM-A, uncovering a previously unreported function for this bacterial protease. These findings propose a novel mechanism for *H. pylori* to disrupt epithelial integrity and functions, breaking new ground in the understanding of the pathogenesis of this highly prevalent and clinically relevant infection.

## Introduction

*Helicobacter pylori* infects half of the human population throughout the world, and persistent colonization of this bacterium increases the risk for diseases such as peptic ulcer and gastric cancer.^1^ *H. pylori* colonizes the mucus that overlies the gastric epithelium and is able to adhere to epithelial cells, with a particular tropism for cell-cell junctions.^2,3^

The most apical set of intercellular junctions are the tight junctions that act as a barrier to pathogen entry into deeper tissues. Tight junctions also control paracellular permeability across the epithelium and serve as a barrier to intramembrane diffusion of components between the apical and basolateral membrane domains.^4^ They are formed by various transmembrane proteins and by cytosolic proteins that connect the former to the cytoskeleton and to different types of signaling proteins.^5^ In epithelial cells, the transmembrane protein junctional adhesion molecule A (JAM-A), also known as F11R, has been implicated in the regulation of the barrier and in cell polarity, adhesion, migration, and invasion.^6–9^ The human JAM-A contains two immunoglobulin (Ig)-like loops in the extracellular domain, a single transmembrane domain, and a short 40-amino-acid cytoplasmic tail.^10^ The C-terminus of JAM-A contains a PDZ domain-binding motif responsible for interactions with cytoplasmic adaptors, including ZO-1/2, Afadin, and PAR3.^11–14^

As part of their pathogenesis, numerous microorganisms, including *H. pylori*, have developed strategies to overcome the tight junctional defense barrier, allowing them to survive, persist, and induce pathology in their hosts.^15^ Several lines of evidence show that *H. pylori* is able to disrupt the structure and functions of tight junctions. Electron microscopy studies of infected individuals have detected *H. pylori* in intercellular spaces below the tight junctions on the basolateral side of the cells and in deeper sites near the lamina propria, thus showing that the bacteria are able to disrupt the tight and adherens junctions.^16^ *In vivo* models have shown that *H. pylori* infection is linked to gastric mucosal barrier dysfunction,^17–19^ and eradication of the infection in human subjects is followed by a decrease in gastric permeability.^20,21^

The *H. pylori* virulence factor CagA has been associated with displacement of tight junction proteins from cell-cell contacts,^22^ which may occur by interaction with ZO-1 and JAM-A, recruiting them to the site of bacterial attachment.^23^ CagA may also interact with PAR1/MARK, leading to altered cell polarity.^24^ Recently, it was shown that the serine protease HtrA secreted by *H. pylori*, in addition to cleaving E-cadherin, also cleaves the extracellular domains of occludin and claudin-8, thereby allowing the bacteria to deliver CagA basolaterally in the membrane.^25,26^

Here, we provide evidence that *H. pylori* cleaves the cytoplasmic domain of JAM-A, compromising gastric epithelial barrier function and cell-cell adhesion. Moreover, we identify PqqE as the *H. pylori* protease that cleaves JAM-A. Our findings reveal a novel mechanism that *H. pylori* uses to disrupt the structure and function of epithelial tight junctions, which may contribute to bacterial pathogenesis.

## Results

### H. pylori disrupts the tight junction protein JAM-A in gastric epithelial cell lines, primary cells, and gastric biopsy specimens

**Figure 1.**
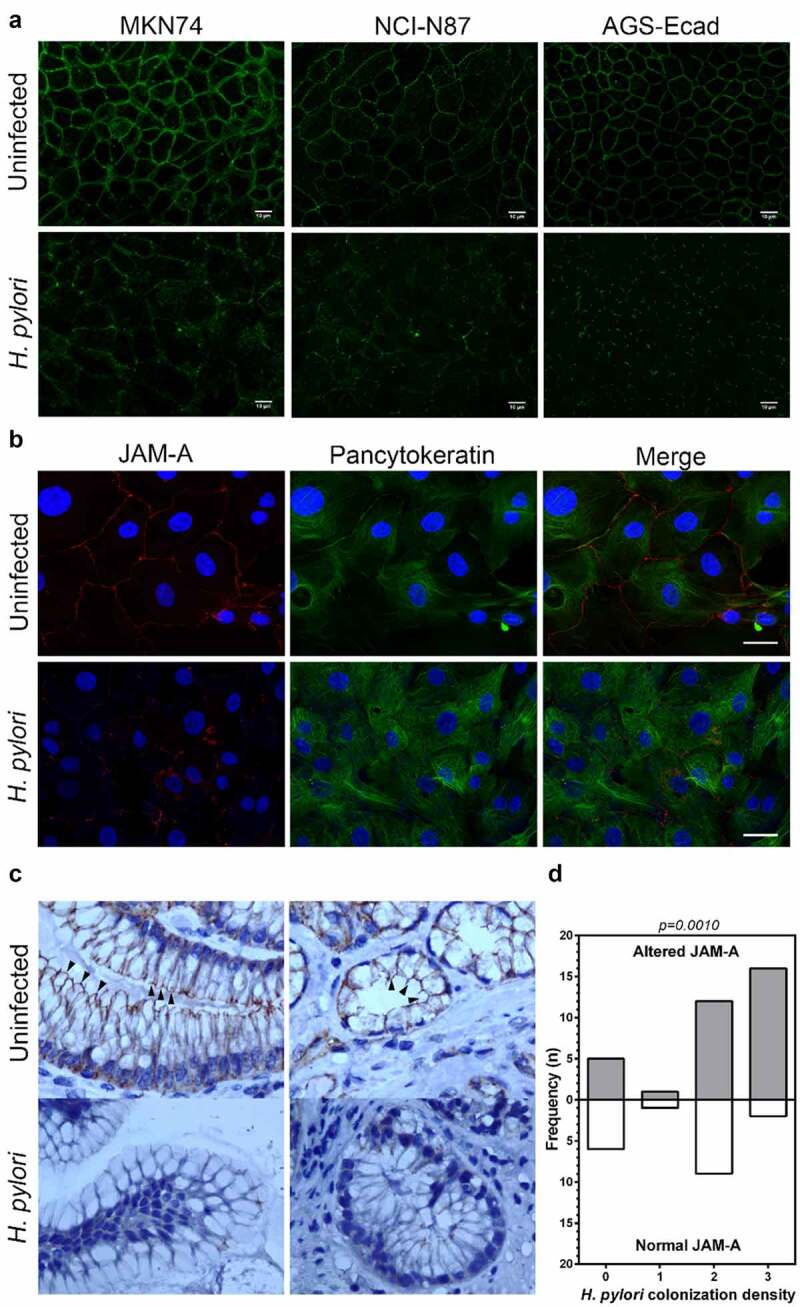
*H. pylori* disrupts the tight junction protein JAM-A in gastric epithelial cell lines, primary cells, and gastric biopsy specimens. (A) JAM-A immunofluorescence (green) in uninfected and *H. pylori* 26695-infected gastric cell lines MKN74, NCI-N87, and AGS-Ecad; scale bar, 10 μm; n = 3. (B) JAM-A immunofluorescence (red) in uninfected and *H. pylori* 26695-infected human primary gastric epithelial cells. A pancytokeratin (green) antibody was used to confirm the epithelial origin of the cells. Nuclei were counterstained with DAPI (blue); scale bar, 10 μm; n = 3; (C) JAM-A immunohistochemistry in human gastric biopsies specimens of *H. pylori*-infected and uninfected patients. Normal JAM-A pattern is a continuous staining circumscribing the apical region of epithelial cells (black arrowheads, upper panels). Altered JAM-A is the interruption of the belt pattern, or loss of expression (lower panels). Pictures were originally obtained with a 1000x magnification. (D) Frequency of JAM-A alterations in 52 gastric biopsy specimens according to the *H. pylori* colonization density (0, absent; 1, mild; 2, moderate; and 3 marked). The two-sided Mann-Whitney U test was used to determine statistical significance

Because *H. pylori* prefers colonizing close to tight junctions^3^ and can impair their functions,^27^ we assessed the impact of *H. pylori* strain 26695 on JAM-A in a panel of gastric cell lines. We used MKN74, NCI-N87, and AGS cells stably transduced with wild-type E-cadherin (AGS-Ecad), which are all able to form competent adherens and tight junctions^27,28^ (Supplementary Figure S1). Immunofluorescence studies showed that in uninfected cell monolayers, JAM-A was localized at the cell membrane, in a typical honeycomb-like pattern. By contrast, after infection with *H. pylori* 26695, there was a decrease of JAM-A expression at the membrane and a delocalization of the protein to the cytoplasm (Figure 1A). This result was observed in all cell lines, thus indicating an effect independent of the cell line model used. No alterations in JAM-A mRNA expression were observed upon *H. pylori* infection (Supplementary Figure S2).

To investigate whether our observations could be recapitulated in primary cells, we used short-term cultures of primary human gastric epithelial cells for infections.^29^ After confirming the epithelial origin of gastric cells with an anti-pancytokeratin antibody, we observed that uninfected cells displayed JAM-A at the cell-cell contacts, while 24 hours after infection with *H. pylori*, the surface expression of JAM-A was strongly reduced (Figure 1B).

To determine the impact of *H. pylori* infection on JAM-A expression *invivo*, we performed immunohistochemistry in 52 gastric biopsy specimens of *H. pylori*-infected (n = 41) and non-infected (n = 11) patients. In normal biopsy specimens, JAM-A was present as a continuous belt, circumscribing the apical region of the epithelial cells. Changes in the expression patterns of JAM-A included the interruption of membrane staining with a loss of the belt pattern and a loss of expression (Figure 1C). In particular, in these specimens, and when we compared the expression patterns of JAM-A among patients, we observed a higher frequency of JAM-A alterations associated with an increase in the density of *H. pylori* colonization (Figure 1D). No statistically significant differences were observed between the histopathological parameters of chronic inflammation or polymorphonuclear activity and altered JAM-A expression.

Altogether, the results obtained in primary cells and in gastric biopsies validate our findings in cell lines and strongly support the notion that *H. pylori* infection alters JAM-A *invivo*.

### H. pylori cleaves the cytoplasmic domain of JAM-A

**Figure 2.**
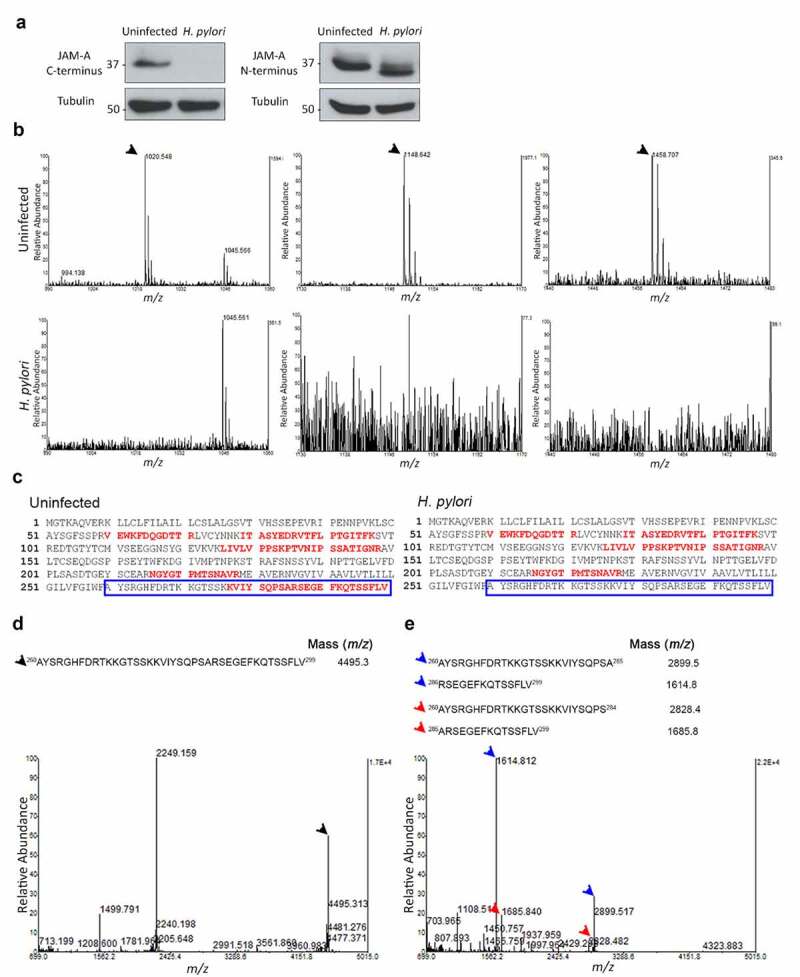
*H. pylori* cleaves the cytoplasmic domain of JAM-A. (A) Western blots of JAM-A in uninfected or *H. pylori* 26695-infected AGS-Ecad cells using antibodies against the cytoplasmic C-terminus and the extracellular N-terminus domain of the protein. Data are representative of n ≥ 4 experiments; (B) MALDI-TOF mass spectrometry analysis of N-terminus immunoprecipitated JAM-A (from *H. pylori* 26695-infected and non-infected samples) after separation in SDS-PAGE. Mass spectra were obtained after in gel tryptic digestion of excised bands and peptide peaks related to JAM-A cytoplasmic domains are indicated by arrows with the following correspondence m/z 1020.548 (VIYSQPSAR), m/z 1148.642 (KVIYSQPSAR), and m/z 1458.707 (SEGEFKQTSSFLV). This experiment was performed twice; (C) Protein coverage data from the uninfected and *H. pylori*-infected samples. Tryptic peptide fragments obtained are depicted in red in the full protein sequence of JAM-A. The JAM-A cytoplasmic domain is identified by the blue boxes. (D and E) MALDI-TOF mass spectrometry analyses of a synthetic peptide of the full cytoplasmic domain of JAM-A incubated with ultra-pure water (D) or with *H. pylori* 26695 sonicate (E). Results are representative of n ≥ 5 experiments, each with similar results. Arrows indicate the peak which corresponds to the full peptide (black), and the peaks obtained after incubation with *H. pylori* (blue, major pair of peptides; red, minor pair of peptides)

To better elucidate the nature of the JAM-A alteration induced by *H. pylori* infection, we conducted western blot analyses with two antibodies, recognizing epitopes either at the extracellular N-terminus or at the cytoplasmic C-terminus regions of this tight junction protein. Using the antibody directed against the cytoplasmic domain of JAM-A, and after infection of cell monolayers with *H. pylori*, we observed a significant decrease in the detection of the protein (Figure 2A). Interestingly, the use of the antibody directed against the extracellular region of JAM-A detected a lower-molecular-weight JAM-A protein in *H. pylori*-infected cells. These results suggest that cytoplasmic cleavage is the most likely phenomenon for the appearance of a lower-molecular-weight JAM-A. Additional experiments incubating lysates of different gastric cell lines and of *H. pylori* led to equivalent results, suggesting that the factor responsible for this cleavage is bacterial-associated and independent of the cell line used (Supplementary Figure S3A). Similar infection experiments with *Campylobacter jejuni, Campylobacter coli*, and *Escherichia coli* suggested that this phenotype is *H. pylori*-specific (Supplementary Figure S3B).

To confirm that *H. pylori* cleaves JAM-A, we performed MALDI-TOF/TOF mass spectrometry. Briefly, JAM-A from uninfected and infected cells was immunoprecipitated with the extracellular domain antibody and then separated by SDS-PAGE. JAM-A protein bands corresponding to the full-length (flJAM-A; in non-infected cells) and to the lower-molecular-weight JAM-A (sJAM-A; in *H. pylori*-infected cells) were excised and in gel digested with trypsin. After mass spectra acquisition of the resulting peptides, both flJAM-A and sJAM-A proteins were confirmed by Peptide Mass Fingerprint (PMF).

The flJAM-A peptidic mass spectrum was compared to that of the sJAM-A, and while in flJAM-A we identified three peptide peaks associated with the cytoplasmic C-terminus at m/z 1020.5, 1148.6, and 1458.7, in sJAM-A we could not identify any peptide peak associated with the JAM-A cytoplasmic domain (Figure 2B and C and Supplementary Tables S1, S2, and S3). These results confirm that the lower-molecular-weight sJAM-A detected by western blot with the antibody against the extracellular domain of the protein in the infections of gastric cells is the result of the cytoplasmic cleavage of JAM-A by *H. pylori*.

To determine the JAM-A cleavage site, we performed an *invitro* cleavage assay followed by MALDI MS analysis. A 40-amino-acid synthetic peptide corresponding to the full cytoplasmic domain of JAM-A, matching amino acids 260 to 299 in the human protein sequence (UniProtKB accession number Q9Y624), was incubated with either *H. pylori* sonicates or ultra-pure water (control). In the control condition, the peptide peak was observed at m/z 4495.3 (Figure 2D). The incubation of the peptide with sonicates of *H. pylori* 26695 resulted in two pairs of peptides: a major pair at m/z 2899.5 and 1614.8, and a minor pair at m/z 2828.4 and 1685.8 (Figure 2E). These results suggest that JAM-A cleavage by *H. pylori* occurs after Ala_285_ or, to a lesser extent, before Ala_285_.

After determining the cleavage site, we addressed cleavage specificity using three synthetic peptides corresponding to amino acids 277 to 292 of JAM-A, one wild-type control peptide (_277_KVIYSQPSARSEGEFK_292_), and two peptides with substitutions in the amino acids at the cleavage site (_277_KVIYSQPSAKSEGEFK_292_ and _277_KVIYSQPSAGSEGEFK_292_). After incubation of the peptides with *H. pylori* sonicates, cleavage products were detected only for the wild-type control Arg_286_ JAM-A peptide and not for the peptides with Lys_286_ or Gly_286_ substitutions (Supplementary Figure S4). This confirmed the specificity of the cleavage site.

### H. pylori-mediated JAM-A cleavage leads to altered tight junction functions

**Figure 3.**
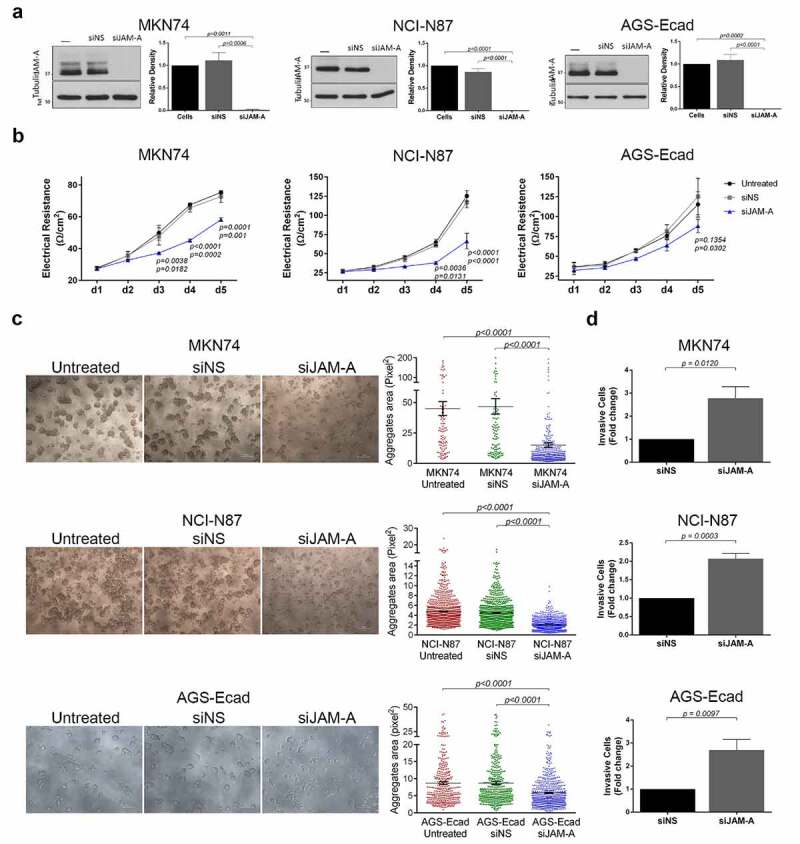
JAM-A contributes to epithelial barrier function, cell-cell adhesion, and suppression of cell invasion in the gastric context without *H. pylori* infection. Gastric MKN74, NCI-N87, and AGS-Ecad cells were left untreated, or were transfected with a non-silencing siRNA (siNS), or a siRNA targeted against JAM-A (siJAM-A). (A) Western blots and respective quantification of JAM-A expression to evaluate knockdown efficiency. Data has a normal distribution; the one-way ANOVA with Tukey’s post-hoc test was used to test for significance (n = 3 for MKN74 and AGS-Ecad cells; n = 5 for NCI-N87 cells). (B) Transepithelial electrical resistance (TER) measurements of gastric cell monolayers were conducted for 5 consecutive days. The data has a normal distribution; the two-way ANOVA with Tukey’s post-hoc test was used to test for significance (n = 3 for all cell lines). (C) Cell-cell adhesion properties of gastric cell lines was evaluated by the slow aggregation assay. Representative images of gastric cell aggregates (100x magnification) and respective quantification of their area (pixel^2^) are shown. Each dot represents the size of an individual cell aggregate (scale bar 100 µm). Data does not follow a normal distribution; the Kruskal-Wallis test was used to test for significance (n = 3 for all cell lines). (D) Cell invasion capacity on matrigel-coated transwells is plotted. Data follows a normal distribution; the two-sided unpaired *t* test was used to test for significance (n = 4). Data are represented as means ± s.e.m

Before investigating the functional consequences of *H. pylori*-mediated JAM-A cleavage, we assessed the functions of JAM-A in the gastric context. For that, MKN74, NCI-N87, and AGS-Ecad cells were transfected with a siRNA targeting JAM-A mRNA (siJAM-A) or with a non-silencing siRNA scramble control (siNS), and successful JAM-A knockdown was confirmed by western blot (Figure 3A). Because epithelial JAM-A was shown to play a role in the regulation of the tight junction barrier function,^6^ we evaluated the transepithelial electrical resistance (TER) of gastric cell monolayers. JAM-A downregulation significantly reduced the TER of gastric cell monolayers compared to the TER of scramble siRNA-transfected or untreated cells (Figure 3B). We next evaluated the role of JAM-A in gastric cell-cell adhesion by quantitatively assessing the area of aggregates formed in slow aggregation assays. Significantly smaller aggregates were identified in MKN74, NCI-N87, and AGS-Ecad cells in which JAM-A expression was silenced than in scramble siRNA-transfected or untreated cells (Figure 3C). Since JAM-A loss decreased gastric cell-cell adhesion, we tested its role in cell invasion through matrigel-coated filters. JAM-A silencing significantly increased cell invasion by 2- to 3-fold in comparison to the respective non-silencing siRNA-transfected control cells (Figure 3D). These results show that in the gastric context without *H. pylori* infection, JAM-A is important in the maintenance of the epithelial barrier, in cell-cell adhesion, and in suppressing cell invasion.

**Figure 4.**
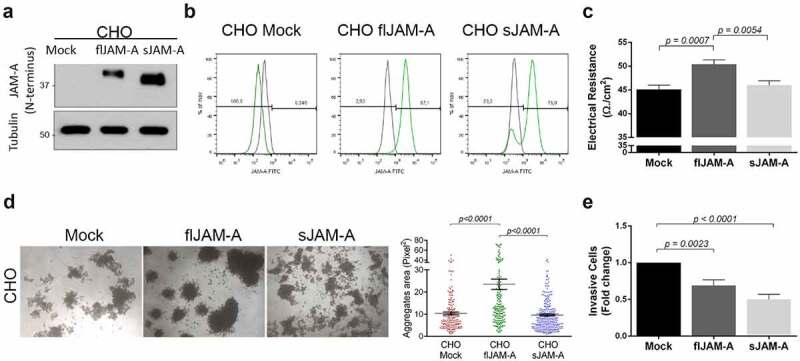
*H. pylori*-mediated JAM-A cleavage leads to altered tight junction functions. CHO cells were stably transfected with the human full-length (flJAM-A) or with JAM-A lacking the sequence cleaved by *H. pylori*, corresponding to the region encoding amino acids 285 to 299 (sJAM-A). (A) Representative western blot using an anti-JAM-A antibody (N-terminus epitope). (B) Flow cytometry using an anti-JAM-A antibody (N-terminus epitope); Green, JAM-A; Grey, IgG negative control (n = 3). (C) Transepithelial electrical resistance (TER) measurements of cell monolayers on day 15. Data follows a normal distribution; the one-way ANOVA with Tukey’s post-hoc test was used to test for significance (n = 3). (D) Cell-cell adhesion properties were evaluated by the slow aggregation assay. Representative images of cell aggregates (40x magnification) and the respective quantification of their area (pixel^2^). Each dot represents the size of an individual cell aggregate (scale bar 100 µm). Data does not follow a normal distribution; the Kruskal-Wallis test was used to test for significance (n = 3). (E) Results are plotted to determine the cell invasion capacity on matrigel-coated transwells. Data follows a normal distribution; the one-way ANOVA with Tukey’s post-hoc test was used to test for significance (n = 6). Data are represented as means ± s.e.m

We next sought to examine the functional consequences of *H. pylori*-mediated JAM-A cleavage. For that, we used CHO cells, which do not endogenously express JAM-A, stably transfected with the human full-length (fl)JAM-A, or with the human JAM-A lacking the sequence corresponding to the region encoding amino acids 285 to 299 (sJAM-A), equivalent to the cleavage induced by *H. pylori*. Cells expressing flJAM-A or sJAM-A were selected by serial bead sorting and the expression of JAM-A was confirmed by western blot and flow cytometry (Figure 4A and B and Supplementary Figure S5). In comparison to cells expressing the flJAM-A, those expressing the sJAM-A had significantly reduced TER (Figure 4C), showing that *H. pylori*-mediated JAM-A cleavage may compromise the epithelial barrier. Furthermore, the quantification of the area of the cell aggregates in slow aggregation assays revealed that cells expressing the flJAM-A formed larger aggregates than did cells expressing the sJAM-A (Figure 4D). This demonstrates the importance of the C-terminal region of JAM-A to cell-cell adhesion. No significant differences were observed in the invasive capacity between flJAM-A and sJAM-A cells (Figure 4E), suggesting that suppression of cell invasion by JAM-A is not influenced by the cleavage of the C-terminus of the protein. Overall, these results show that alterations to the gastric epithelial barrier and to cell adhesion are directly attributable to the lack of a C-terminal portion of JAM-A, which is cleaved upon infection with *H. pylori*.

### Identification of the H. pylori factor that cleaves JAM-A

**Figure 5.**
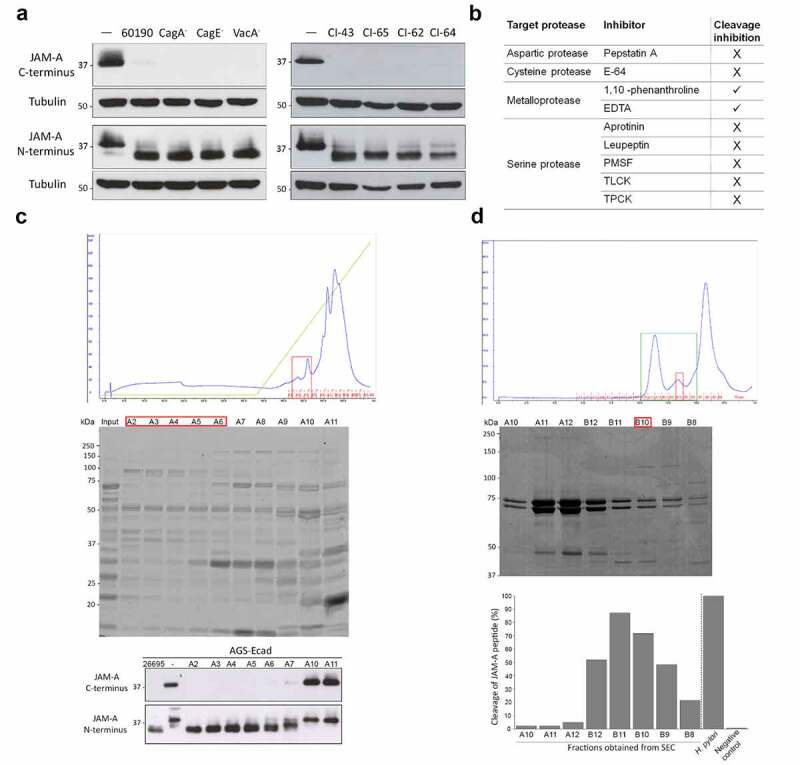
Identification of the *H. pylori* factor that cleaves JAM-A. (A) Western blot of JAM-A in AGS-Ecad cells infected with *H. pylori* 60190, 60190CagA^−^, 60190CagE^−^, or 60190VacA^−^ mutants, or with clinical isolates using antibodies to detect the C- or N-terminus of JAM-A; tubulin was used as loading control. (B) Screening of the type of protease involved in JAM-A cleavage with a panel of protease inhibitors. Cleavage was evaluated by MALDI-TOF/TOF analysis using a cytoplasmic domain JAM-A peptide (_277_KVIYSQPSARSEGEFK_292_) incubated with *H. pylori* 26695 lysates in the presence of the inhibitors. ✓, cleavage inhibition; X, no cleavage inhibition. Experiments were performed n ≥ 2, with similar results. c) *Upper panel*, representative chromatogram obtained after separation of the 100 mM NaCl fraction from Q-Sepharose on SOURCE-15Q. Red boxes, fractions (A2 to A6) selected for size exclusion chromatography (SEC). *Middle panel*, Coomassie blue-stained SDS-PAGE gel of separated fractions. *Bottom panel*, detection of the *H. pylori* protease by western blot after incubation of the fractions with AGS-Ecad cell lysates. *H. pylori* 26695 and uninfected cell lysates were used as positive and negative controls, respectively. (D) *Upper panel*, representative chromatogram obtained from SEC after separation of fractions A2 to A6 obtained from SOURCE-15Q. Green box, fractions (A10 to B8) selected for the detection of cleavage activity; Red box, fraction selected for protein identification. *Middle panel*, Coomassie blue-stained SDS-PAGE gel of separated fractions. *Bottom panel*, MALDI MS analyses for the detection of protease activity of fractions A10 to B8 using a synthetic peptide of the full cytoplasmic domain of JAM-A. *H. pylori* 26695 sonicate and ultra-pure water were used as positive and negative controls, respectively. Protease activity was calculated as the ratio between the sum of the intensity of the two pairs of peaks in the test condition and in the positive control condition. (C and D). Data are representative of three independent experiments, each with similar results

To investigate the extent to which JAM-A cleavage was associated with known *H. pylori* virulence factors, we started by performing a series of experiments with an additional strain (*H. pylori* 60190) and its *cagE* (which does not form a functional T4SS), *cagA*, and *vacA* mutants. As initially observed for strain 26695, *H. pylori* 60190 and all of the mutants were able to induce JAM-A cleavage, as shown by the lost (or significantly reduced) JAM-A detection using the C-terminus antibody, and by the detection of the lower-molecular-weight JAM-A, using the N-terminus antibody (Figure 5A). Additional experiments with *H. pylori* clinical isolates showed that all tested isolates were able to cleave JAM-A (Figure 5A). These results suggest that JAM-A cleavage occurs independently of CagA, or VacA, thus supporting the notion that another *H. pylori* factor is associated with this phenotype and that the T4SS is not used for its delivery.

The next set of experiments was aimed at characterizing the class of proteases involved in *H. pylori*-mediated JAM-A cleavage. It consisted of experiments with a panel of inhibitors of different classes of proteases. A decrease in the *H. pylori*-mediated cleavage activity of the JAM-A peptide was observed when ethylenediamine tetraacetic acid (EDTA) or O-phenanthroline was incubated with *H. pylori* lysates, suggesting that the protease of interest was a metalloprotease (Figure 5B and Supplementary Figure S6).

To identify the *H. pylori* protease that cleaves JAM-A, we combined ion exchange chromatography (IEX), size exclusion chromatography (SEC), and protein identification by mass spectrometry (Figure 5C and D and Supplementary Figure S7A). Briefly, proteins from *H. pylori* 26695 lysates were separated by IEX using a discontinuous NaCl gradient. Collected unbound, 100 mM, 200 mM, and 300 mM eluates were assessed for their ability to cleave JAM-A in total lysates of AGS-Ecad cells by western blot with anti-JAM-A antibodies recognizing the N- and C-terminus. As a positive control, we used a total lysate of *H. pylori* 26695. The 100 mM eluate, which had the highest JAM-A proteolytic activity (Supplementary Figure S7B), was collected and separated in a second IEX. Eluate fractions were assessed for their ability to cleave JAM-A by western blot, as described above (Figure 5C). In fractions A2 to A6, we observed complete proteolytic activity against JAM-A, as the antibody recognizing the C-terminal epitope could not detect JAM-A; concomitantly, the N-terminal antibody detected a smaller molecular weight protein. Fractions A2 to A6 were then pooled together, concentrated, and further purified by SEC (Figure 5D). SEC fractions were incubated with the 40-amino-acid cytoplasmic domain JAM-A peptide and assessed for cleavage activity by MS. The mass spectra for cleavage of the JAM-A peptide obtained for each fraction can be seen in Supplementary Figure S7C. JAM-A proteolytic activity was expressed as the sum of the intensities of the peaks of the two pairs of peptides, which also indicated loss of the peptide through a specific cleavage pattern. Fractions B10 and B11 had the highest activity against the JAM-A peptide (Figure 5D), suggesting that they were enriched in the bacterial protease. In these fractions, we noticed the presence of a new band below 50 kDa, which could not be identified by PMF with statistical confidence due to low protein abundance. To identify the proteins present in this band, we excised it from the gel after having concentrated fraction B10 (Supplementary Figure S7D). In this band, we identified a mixture of protease PqqE (UniProtKB accession no. O25656; encoded by *HP1012*), processing protease YmxG (accession no. O25371; encoded by *HP0657*), and fumarate hydratase class II (accession no O25883; encoded by *fumC*) by PMF. Peptide MS/MS sequencing allowed us to confirm the identities of *H. pylori* proteases PqqE and YmxG (Supplementary Figure S7E and S7F, and Supplementary Tables S4 and S5).

### PqqE is the H. pylori protease that cleaves JAM-A

**Figure 6.**
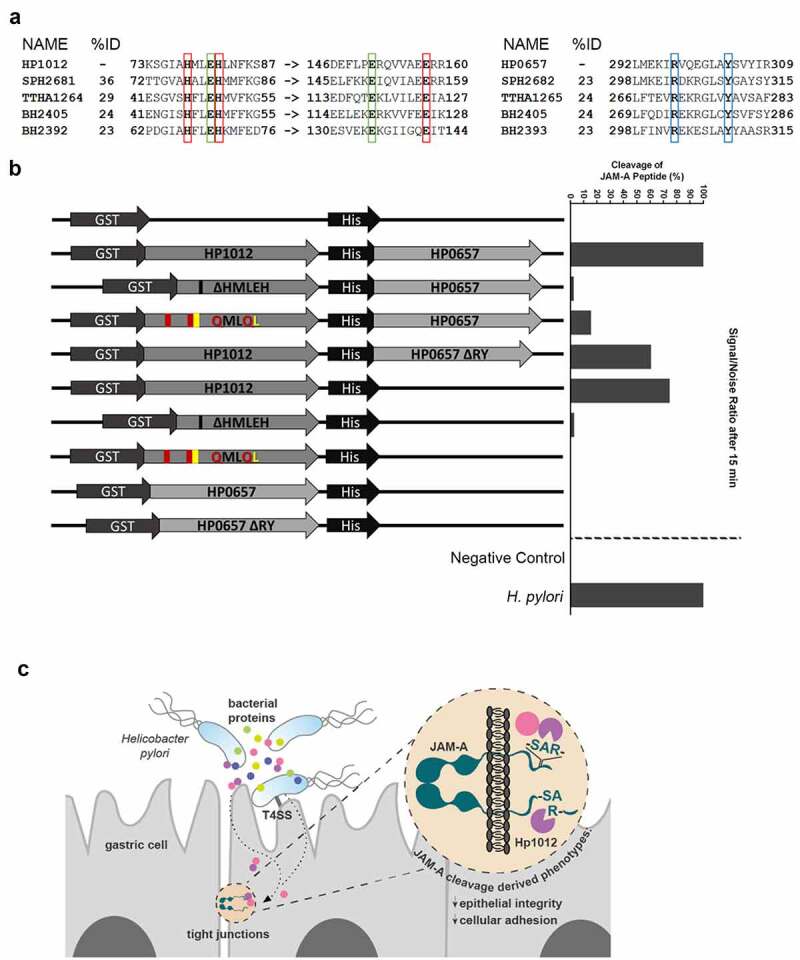
PqqE is the *H. pylori* protease that cleaves JAM-A. (A) Sequence alignments of PqqE/HP1012 and of YmxG/HP0657 with prokaryotic M16B metalloproteases, showing selected fragments that include the metal ligand and active sites, and the R/Y pair. The Zinc-binding motifs (red boxes) and active site (green boxes) are highly conserved across prokaryotic M16B metalloproteases, although they share only a maximum of 36% total homology to HP1012. The R/Y pair (blue boxes) is also highly conserved, sharing a maximum of 24% total homology to HP0657. With the exception of BH2405 from *Bacillus halodurans* that exists as a homodimer, the remaining protein pairs form, or are predicted to form functional heterodimers (SPH2681/SPH2682 from *Sphingomonas sp*. A1; TTHA1264/TTHA1265 from *Thermus thermophiles*; and BH2392/BH2393 from *B. halodurans*). %ID, protein sequence homology. (B) Schematic representation of the constructs used for recombinant protease expression and the respective quantification of the cleavage of the full JAM-A cytoplasmic domain peptide by MALDI MS analysis. Cleavage activity was calculated as the ratio between the sum of the intensity of the two pairs of peaks in the test condition and in the positive control condition. *H. pylori* 26695 sonicate and ultra-pure water were used as positive and negative controls, respectively. (C) Model of *H. pylori*-mediated JAM-A cleavage by PqqE/HP1012

Because fumarate hydratase does not have protease activity, and because our pre-screening with protease inhibitors pointed to the involvement of a *H. pylori* metalloprotease in JAM-A cleavage, we pursued PqqE and YmxG, which belong to the M16B subfamily of metallopeptidases.^30^ Like the other metalloproteases in this subfamily that contain an HXXEH (His-Xaa-Xaa-Glu-His, where Xaa is any amino acid) motif, PqqE/HP1012 has an HMLEH motif that is necessary for zinc binding and catalytic activity. An additional zinc-binding glutamate is also present at a more C-terminal region. YmxG/HP0657 is a non-peptidase homologue, which lacks the HXXEH motif and contains an R/Y pair at a more C-terminal region. Sequence alignment with other known prokaryotic M16B peptidases showed that PqqE/HP1012 has complete conservation of all the residues involved in zinc-binding and catalytic activity, and that YmxG/HP0657 has conservation of the R/Y pair (Figure 6A).

M16B prokaryotic peptidases may form homo or heterodimers.^31^ A heterodimeric M16 peptidase formed by SPH2681 and SPH2682 was described in *Sphingomonas sp*. strain A1, the former containing the HXXEH motif and the latter lacking this motif but containing the R/Y pair.^32^ Taking this into consideration, we next predicted protein interactions for *H. pylori* 26695 PqqE using the STRING database. The results showed that PqqE and YmxG are the most probable functional partners, with a score of 0.997 (Supplementary Table S6).

To demonstrate the involvement of these *H. pylori* proteins in JAM-A cleavage, we cloned *HP1012* from strain 26695 fused to the glutathione-S-transferase (GST) gene and expressed it in *E. coli* BL21 as a GST fusion protein (Supplementary Figure S8), for purification by affinity chromatography in non-denaturing conditions. In addition to wild-type HP1012, recombinant variants lacking the HMLEH motif (ΔHMLEH) or with amino acid substitutions in this motif (QMLQL) were also generated.

Additionally, we cloned *HP0657* fused to a 6-histidine tag and a variant lacking the region that contains the R/Y pair (ΔRY; lacking amino acids G_285_ to Y_307_), alone or downstream *HP1012* or its ΔHMLEH and QMLQL variants (Figure 6B). All recombinants were incubated with the full JAM-A cytoplasmic domain peptide, and peptide cleavage was monitored by MS (Supplementary Figure S9).

The combined HP1012-HP0657 recombinant cleaved JAM-A to an extent similar to that of the control *H. pylori* 26695 strain (Figure 6B). HP1012 alone could also cleave JAM-A, though to a lesser extent than when combined with HP0657. The ΔHMLEH and QMLQL HP1012 variants, alone or in combination with wild-type HP0657, exhibited null or very low activity in cleaving JAM-A. HP0657 and its ΔRY variant also could not cleave the JAM-A peptide.

Overall, these results show that PqqE is the *H. pylori* protease that cleaves the cytoplasmic domain of JAM-A and suggest that YmxG may contribute to the full activity of PqqE.

## Discussion

Here, we demonstrate that *H. pylori* infection leads to the disruption of the epithelial JAM-A *invitro* and *invivo*. The alteration in JAM-A mediated by *H. pylori* occurs through cleavage of the cytoplasmic domain of the protein, for which the cleavage recognition sequence and specificity have herein been determined. JAM-A cytoplasmic cleavage by *H. pylori* is functionally reflected in the loss of the epithelial barrier and decreased cell-cell adhesion. Finally, we uncover the identity of the *H. pylori* protease that cleaves JAM-A, highlighting a previously unreported function of PqqE/HP1012 (Figure 6C).

Previous studies have shown that *H. pylori* is able to disrupt the structure of tight junctions, inducing mislocalization or decreased protein expression of some tight junctional transmembrane components in *invitro*^17,27,33^ and *ex vivo*^22^ infection studies. In our study, we evidenced that *H. pylori* infection leads to the disturbance of the transmembrane tight junction protein JAM-A in multiple junctional-proficient gastric epithelial cell lines, as well as in human primary gastric epithelial cells. Furthermore, we demonstrated that JAM-A alterations also occur in the gastric epithelium of *H. pylori*-infected patients, emphasizing that this process also takes place *invivo*.

Our initial investigations of the nature of the JAM-A alteration induced by *H. pylori* that relied on the use of antibodies recognizing either the extracellular or cytoplasmic domains of the protein, were indicative of a cytoplasmic cleavage by *H. pylori* – a phenomenon that was not replicated in infections with *C. jejuni, C. coli*, and *E. coli*. Confirmation that *H. pylori* cleaves the cytoplasmic domain of JAM-A was supported by MALDI-TOF/TOF mass spectrometry analyses, which could not identify peptide peaks associated with the JAM-A intracellular domain in *H. pylori*-infected cells. Experiments using synthetic peptides of the cytoplasmic domain of JAM-A were key to identifying the fact that cleavage occurs at the _284_SerAlaArg_286_ motif, preferentially after Ala_285_.

Functionally, epithelial JAM-A is implicated in a diverse array of processes, including regulation of the barrier function, cell adhesion, migration and polarity, and suppression of cell invasion.^8,12,14,34^ Our data generated by the siRNA-mediated silencing of JAM-A expression in different cell lines confirmed that, in the gastric setting, JAM-A plays a role in the maintenance of the epithelial barrier and contributes to cell-cell adhesion and the suppression of cell invasion.

Functional characterization of the effect of *H. pylori*-mediated JAM-A cleavage was addressed in CHO cells engineered to express either the full-length JAM-A or a shorter JAM-A lacking the last 14 amino acids. Our findings showed that *H. pylori*-mediated cytoplasmic cleavage of JAM-A led to the loss of epithelial barrier function and decreased cell-cell adhesion, suggesting that the cleaved region is critical to the maintenance of these functions. Central to the biological activities of JAM-A, the cytoplasmic C-terminus of the protein contains a PDZ domain-binding sequence in residues _295_SerSerPheLeuVal_299_, which is important for direct interactions with PDZ-domain-containing scaffolding and signaling proteins such as ZO-1 and PAR3.^10^ These interactions will likely be affected by JAM-A cleavage induced by *H. pylori*. Furthermore, the cytoplasmic domain of JAM-A contains a phosphorylation site at Ser_284,_^35^ which is adjacent to the *H. pylori* cleavage site. Ser_284_ phosphorylation is actually critical for epithelial JAM-A functions, as phosphorylation-deficient JAM-A cells fail to mature cell-cell contacts and have an impaired barrier function.^35^ Taken together, these features might explain the functional consequences observed in the context of *H. pylori*-associated cytoplasmic JAM-A cleavage.

We identified M16B metalloprotease subfamily PqqE/HP1012 and YmxG/HP0657 in the *H. pylori* fraction that cleaved the cytoplasmic JAM-A domain. Heterodimeric M16B proteases are formed by a catalytically active β-subunit with an active site and metal-ligand site and with a non-peptidase α-subunit that lacks the active and metal-ligand sites.^31^ In addition to the fact that this description fits with those of *H. pylori* PqqE and YmxG, *in silico* analysis predicted PqqE and YmxG as the most probable functional partners.

Experiments addressing JAM-A cytoplasmic domain cleavage by incubation of recombinant bacterial proteases with a synthetic peptide showed that YmxG alone could not cleave JAM-A, consistent with its description of a non-peptidase.^30^ While recombinant PqqE cleaved the JAM-A cytoplasmic peptide, recombinants with deletions or amino acid substitutions of the metal ligand-active site had almost completely abolished protease activity. Noteworthy was the fact that while recombinant PqqE alone had a proteolytic activity of about 70%, the PqqE-YmxG recombinant led to full cleavage of JAM-A, similar to what was observed for the positive control *H. pylori*. This suggests that the combination of the two proteins is necessary for a fully functional protease complex. The R/Y pair in the non-peptidase α-subunit of heterodimeric M16B proteases is functionally important for the catalytic activity of the β-subunit.^31^ Accordingly, the PqqE-YmxGΔRY recombinant displayed lower levels of JAM-A cleavage in comparison to the double recombinant PqqE-YmxG. Although the substrate specificity of the M16B protease subfamily is diverse, the cleavage sites are near the terminal protein residues, preferably at Xaa-Arg bonds,^30,36^ which is compatible with the JAM-A motif cleaved by PqqE.

*HP1012*, the gene that encodes PqqE, appears to be essential in *H. pylori*. Global transposon mutagenesis in strain G27 showed no observable transposon insertions in *HP1012*.^37^ Accordingly, we were also unable to generate *HP1012* mutant strains.

That PqqE cleaves the cytoplasmic domain of JAM-A independently of the T4SS raises the question of how the bacterial protease reaches the inside of the host cell. Bioinformatics predictions and direct mass spectrometry-based methods identified a signal sequence in PqqE proposing its targeting to the outer membrane or secretion.^38,39^ PqqE was reported in the extracellular proteome of *H. pylori*.^40^ Analysis of the composition of the *H. pylori* exoproteome at multiple phases of bacterial growth identified PqqE as enriched in the culture supernatant compared to subcellular fractions derived from intact bacteria. This suggests the selective release of these proteins into the extracellular space. In fact, PqqE was the second-highest enriched protein in the supernatant after HtrA.^39^ PqqE can also be found in the proteome of outer membrane vesicles (OMVs) of *H. pylori*.^41^ *H. pylori* OMVs can be uptaken by gastric cells by clathrin-dependent and -independent endocytic pathways, depending on the lipid composition and receptor clustering in the host cell membrane.^42^ These vesicles can deliver bacterial components into host cells, including peptidoglycan and the CagA virulence factor.^43,44^ Although the fate of *H. pylori* OMVs inside the host cells is largely unknown, one may speculate they may function as delivery vehicles of the PqqE protease into the host cell cytoplasm, where PqqE is able to access the C-terminus of JAM-A leading to its cleavage.

Knowledge of proteases that affect *H. pylori* pathogenesis is still very limited. Recently, the extracellular cleavage of the transmembrane tight junction proteins occludin and claudin-8 was reported during *H. pylori* infection.^26^ This occurs due to the action of the *H. pylori* HtrA, which also cleaves the extracellular domain of E-cadherin, but not JAM-A,^25^ to promote disruption of cell-cell adhesion, thereby allowing the bacteria to invade the intercellular space and reach integrins for CagA injection.^26^

Tight junctions act as a barrier for pathogens, and yet *H. pylori* has developed strategies to dampen these structures. The degradation of the cytoplasmic domain of a transmembrane junctional protein represents a unique mechanism for *H. pylori* to breach this barrier, which may have a profound impact on bacterial pathogenesis. Finally, the identification of strategies that *H. pylori* uses to hijack the host cell functions may reveal novel candidates for therapeutic interventions in this highly prevalent and clinically relevant infection.

## Experimental section

### Cell cultures

Human gastric epithelial cell lines AGS-Ecad (stably transfected with E-cadherin),^28^ NCI-N87 (obtained from ATCC, Cat# CRL-5822), and MKN74 (a kind gift from Carla Oliveira, Univ. Porto;^45^ and the Chinese hamster ovary (CHO) cell line (a kind gift from Joana Figueiredo, Univ. Porto,^46^ were used. Cells were cultured in RPMI medium (Labclinics), while CHO cells were cultured in Alpha-MEM (Gibco), supplemented with 10% of heat inactivated fetal bovine serum (HyClone®, Thermo Fisher Scientific) and 1% penicillin/streptomycin solution (Gibco). Blasticidin at 0.1% was added to the culture medium for AGS-Ecad and CHO cells. The cells were incubated at 37°C under a 5% CO_2_ humidified atmosphere. The human cell lines were authenticated using the PowerPlex® 16 HS kit (Promega), allowing for the detection of 15 STR loci and of Amelogenin. All cell lines tested negative for mycoplasma contamination.

### Bacteria cultures and infections of cell lines

*H. pylori* strain 26695 (ATCC 700392) and *Escherichia coli* (ATCC 25922) were purchased from ATCC (Rockville). *H. pylori* 60190 (ATCC 49503) and its respective 60190CagA^−^, 60190CagE, and 60190VacA^−^ mutants were obtained from John Atherton (Univ. Nottingham).^47^ *H. pylori* isolates that had a different CagA status from our collection were also used.^48,49^ *Campylobacter jejuni* and *Campylobacter coli* A11 were obtained from Nuno Azevedo (Univ. Porto). *H. pylori* were cultured for 48 hours in tryptic soy agar (TSA) supplemented with 5% sheep blood (Becton Dickinson) and incubated at 37°C under microaerophilic conditions, as previously described.^48^ For liquid media cultures, F12 medium (Gibco) supplemented with 1x cholesterol (Gibco) was used, and bacteria were grown under microaerophilic conditions at 37°C with constant rotation (150 rpm) over 72 hours.

For in vitro infections, gastric cells were grown in an antibiotic-free culture medium. A 48-hour bacteria culture in TSA plates was collected in sterile PBS, pH 7.4, and added to the cells at a multiplicity of infection (MOI) of 100, unless otherwise stated. Infections were carried for 24 hours with *H. pylori* strain 26695, unless otherwise stated.

### Immunofluorescence

Cells were grown on glass coverslips (Marienfeld), fixed with ice-cold methanol, blocked with 5% goat serum, 0.3% Triton X-100 in PBS (pH 7.4) for 1 hour at room temperature, and stained with goat polyclonal anti-JAM-A (AF1103, R&D Systems), mouse monoclonal anti-ZO-1 (33–9100, Thermo Fisher Scientific), or rabbit polyclonal anti-occludin (71–1500, Thermo Fisher Scientific) antibodies. The rabbit polyclonal anti-JAM1 (36–1700, Invitrogen) and the mouse monoclonal anti-cytokeratin pan-FITC (fluorescein isothiocyanate, clone C-11, F3418, Sigma-Aldrich) antibodies were used in short-term primary cell cultures. Coverslips were then washed with PBS pH7.4 and incubated with the respective secondary antibodies ((H + L) Highly Cross-Adsorbed goat anti-rabbit IgG Alexa Fluor 594, goat anti-mouse IgG Alexa Fluor 488, goat anti-rabbit IgG Alexa Fluor 488, and donkey anti-goat IgG Alexa Fluor 488, all Thermo Fisher Scientific). After washing, coverslips were mounted with Vectashield Antifade Mounting Medium with DAPI (Vector Laboratories) and fluorescence was monitored in a Zeiss Axio Imager Z1 Apotome microscope. Fiji (ImageJ) was used for image processing and analysis.

### Quantification of JAM-A/F11R mRNA by real-time quantitative PCR

*F11R* expression was determined using TaqMan Universal PCR Master Mix (Applied Biosystems) and the *F11R* probe assay (Integrated DNA Technologies, USA). Relative *F11R* expression was normalized to levels of glyceraldehyde 3-phosphate dehydrogenase (Human GAPDH Endogenous Control FAM Dye/MGB Probe, Non-Primer Limited, Applied Biosystems).

### Immunohistochemistry and histopathology

Fifty-two formalin-fixed and paraffin-embedded gastric biopsy specimens were retrospectively retrieved from the Department of Pathology of Centro Hospitalar Universitário S. João (CHUSJ). They consisted of diagnostic samples obtained from patients undergoing upper endoscopy due to complaints of dyspepsia (mean age [±SD], 40.5 ± 15.9 years; female-to-male ratio, 1.7:1). Cases were selected for the absence of past/present peptic ulcer disease, atrophy, intestinal metaplasia, dysplasia, and gastric carcinoma. The study was approved by the ethical committee of CHUSJ (205/2010). Specimens were stained with hematoxylin/eosin and modified Giemsa. Histopathological parameters were scored by an experienced pathologist (FC) who was blinded to the patients’ clinical information and who used the criteria described in the Updated Sydney System for the classification of gastritis.^50^ Chronic inflammation, polymorphonuclear activity, and *H. pylori* colonization density were graded on ordinal scales as absent (0), mild (1), moderate (2), or marked (3).

Slides were deparaffinized and rehydrated. After antigen retrieval with Trilogy^TM^ buffer (Cell Marque), at 98°C for 20 minutes, using the PT module (LabVision), slides were blocked with hydrogen peroxide and incubated with the rabbit monoclonal anti-JAM1 (clone EP1042Y) antibody (ab52647, Abcam, 1:100) for 45 minutes. Detection was performed by incubating the sections for 30 minutes with the Poly-HRP-anti-mouse/rabbit/rat IgG polymer (Immuno Vision Technologies). Slides were developed for 5 minutes with cromogenic diamonobenzidine (Cell Marque) and counterstained with Mayer’s hematoxylin. The scoring of immunostained slides was performed by an experienced pathologist. As a positive control, a normal gastric specimen without *H. pylori* infection that showed JAM-A in a continuous belt-like pattern close to the apical region of the epithelial cells in superficial and foveolar epithelium was used. Cases with these characteristics were considered normal and otherwise were classified as having an altered pattern.

### Isolation and ex vivo short-term culture of gastric epithelial cells

Gastric epithelial cells were isolated from biopsies obtained from adult patients undergoing upper endoscopy due to complaints of dyspepsia. The study was approved by the ethics committee of the Instituto Português de Oncologia (IPO) in Porto, and informed consent was obtained from all patients. Isolation and culture of epithelial cells were performed as previously described.^29^ Gastric epithelial cells were cultured in standard culture conditions for 4 days prior to the addition of *H. pylori* (or PBS in control cultures) for another period of 24 hours.

### SDS-PAGE and western blot

Total cell lysates were prepared in a cold lysis buffer (1%NP-40, 1% Triton X-100 in PBS, pH7.4) containing a cocktail of protease (Roche Applied Science) and phosphatase (Sigma-Aldrich) inhibitors. The exceptions were the experiments in which lysates of gastric cells were incubated with *H. pylori* sonicates, which took place in the absence of protease and phosphatase inhibitors. The protein concentration of the cleared lysate recovered upon centrifugation was assessed using the Bio-Rad protein assay kit (Bio-Rad), according to the manufacturer’s protocol. Samples were loaded in 10% or 7.5% SDS-PAGE gels and then stained with BlueSafe (NZYtech) or transferred onto a nitrocellulose membrane (GE Healthcare) blocked in 5% skim milk in PBS pH7.4 with 0.1% Tween-20, for 1 hour at room temperature. Membranes were incubated with rabbit monoclonal anti-JAM1 (clone EP1042Y), rabbit polyclonal anti-JAM1 (36–1700, Zymed), mouse monoclonal anti-tubulin (clone DM1A, T9026, Sigma-Aldrich), mouse monoclonal anti-6x-His (MA1-21315, Thermo Fisher Scientific), or mouse monoclonal anti-GST (MA4-004, Thermo Fisher Scientific). As secondary antibodies, the HRP-conjugated-donkey anti-rabbit IgG (NA934, GE Healthcare) and anti-sheep anti-mouse IgG were used (NA931, GE Healthcare). Detection was performed with Clarity Western ECL Substrate (Bio-Rad). The quantification of band intensities was performed using the Quantity One 1-D analysis software version, 4.6.6 (Bio-Rad).

### Cell transfection

Cell transfection was performed with Lipofectamine® 2000 (Life Technologies) according to the manufacturers’ protocol, using 50 nM of JAM-A siRNA (Hs_F11R_8 FlexiTube siRNA; QIAGEN) and a negative control siRNA (All Stars Negative Control siRNA; QIAGEN). Transfection efficiency was evaluated using western blot.

### Establishment of cell lines stably expressing full JAM-A (flJAM-A) and short JAM-A (sJAM-A)

Stable cell lines with either flJAM-A or sJAM-A were generated in CHO cells, which do not express JAM-A. The human flJAM-A open reading frame (Ultimate™ ORF Clone ID IOH12781_Homo sapiens F11 receptor) and the sJAM-A vector generated through the deletion of the region that encodes Ala_285_ to Val_299_ were obtained from Invitrogen. Both flJAM-A and sJAM-A were cloned into a pENTR™ Directional TOPO® Entry vector. After confirmation of the sequences of both vectors using the M13 primers (Supplementary Table S7), flJAM-A and sJAM-A sequences were cloned in the pEF6/myc-His A expression vector, using the Gateway® cloning technology according to the manufacturer’s instructions.

### Flow cytometry

CHO-Mock, CHO flJAM-A, and CHO sJAM-A cells grown in confluence were washed with PBS pH7.4 and detached with Versene solution (Gibco). After centrifugation, 1 × 10^6^ cells were resuspended and incubated for 30 minutes in a cold blocking solution (5% BSA in PBS). Next, cells were incubated with the mouse monoclonal anti-JAM-A (J10.4) FITC-conjugated antibody (sc-53623, Santa Cruz Biotechnology) or isotype-specific IgG1, on ice for 1 hour, and analyzed in a Coulter® XL-MCL™ (Beckman Coulter Inc) flow cytometer. Data were analyzed using the FlowJo® software.

### Transepithelial electrical resistance (TER) analysis

Gastric epithelial cells were seeded at 100% confluence in 6.5 mm Transwell® with a 3.0 µm Pore Polyester Membrane Insert (Corning), as we described previously.^27^ In brief, a Millicell ERS voltohmmeter (EMDMillipore) was used for evaluating monolayer integrity and procedures were performed according to the manufacturer’s instructions. TER was measured every 24 hours for up to five days, or at the referred time points. Plates were left for 15 minutes to reach room temperature in order to avoid the influence of temperature and the media were changed after each measurement. TER values were expressed in Ohms/cm^2^.

### Slow aggregation assays

Slow aggregation assays were performed as previously described.^27^ Briefly, after dissolving in a microwave 0.67% bacto agar with sterile PBS, pH 7.4, the agar suspension was distributed into 96-well plates (100 µL per well), and plates were left on a horizontal surface, at 4°C. 20,000 cells (or 50,000 cells for the NCI-N87 cell line) suspended in a volume of 200 µL of complete medium, were placed on top of the solidified agar and incubated for 5 days (or 2 days for AGS-Ecad cells). The area of the cell aggregates was measured using the Quantity One software.

### Transwell matrigel invasion assays

Invasion assays were performed as previously described.^27^ Briefly, after hydrating Matrigel-coated 24-well invasion inserts (8 μm pores; Corning) with RPMI, 5 × 10^4^ cells suspended in RPMI with 10% FBS and without antibiotics were placed on the upper side of the insert. The bottom side of the insert was filled with RPMI. After a period of 24 hours of incubation, a cotton swab was used to remove the non-invading cells, whereas the remaining cells were fixed for a period of 10 minutes in methanol on ice. The filter was mounted with Vectashield with DAPI (Vector Laboratories) on a microscope slide. The entire filter was used to score the number of invading cells, at 20X magnification, in a Leica DM2000 fluorescence microscope.

### Preparation of H. pylori sonicates

Bacteria grown in F12 liquid media for 72 hours were collected by centrifugation at 15000xg for 15 minutes at 4°C. A bacterial pellet was suspended in an ice-cold phosphate buffer (20 mM NaH_2_PO_4_.H_2_O, pH 7.4) and lysed by sonication in a Bandelin Sonopuls (BANDELIN electronic) with 9 cycles of 3 pulses of 1 minute, at 90% power. Bacterial debris was then removed by centrifugation and the supernatant was recovered and filtered in a 0.2 µm pore filter (GE Healthcare).

### Protease purification from H. pylori sonicates

Ion Exchange Chromatography (IEX) and Size Exclusion Chromatography (SEC) were used to separate the proteins from the bacterial lysate. IEX was performed using the Q Sephrose Fast Flow (GE Healthcare Life Sciences). The column was equilibrated in 20 mM NaH_2_PO_4_.H_2_O, pH7.4 before the bacterial lysate was added to the column and incubated overnight at 4°C. Bacterial proteins were separated using a discontinuous NaCl gradient on ice-cold 20 mM NaH_2_PO_4_.H_2_O, pH7.4 with 0.1 M, 0.2 M, and 0.3 M NaCl. The fraction obtained from the 0.1 M elution step was dialyzed in a cellulose membrane (Sigma-Aldrich) in 20 mM NaH_2_PO_4_.H_2_O, pH7.4 at 4°C for 2 hours before the next IEX. The second IEX was performed in a Source 15Q 4.6/100 PE column (GE Healthcare). Protein purification was performed with an ÄKTA purifier (GE Healthcare) with a continuous NaCl gradient from 0 to 0.35 M NaCl in 20 mM NaH_2_PO_4_.H_2_O, pH7.4. Proteins were then collected into 1 mL fractions. For SEC, only the fractions from SOURCE-15Q with proteolytic activity tested against the cytoplasmic domain of JAM-A were selected. Samples were concentrated by ultrafiltration using a 10 kDa pore size Amicon (EMDMillipore), followed by separation in a Superdex 200 Increase 10/300 GL column (GE Healthcare). Chromatography was performed in a 150 mM NaCl, 20 mM NaH_2_PO_4_.H_2_O, pH7.4 buffer and 0.5 mL volume fractions were collected.

### Detection of protease activity

The protease activity of IEX and SEC fractions against the JAM-A cytoplasmic domain was detected by western blot or by MALDI-TOF/TOF. For western blot detection, 30 µL of each selected fraction was incubated for 16 hours at 37°C with 10 µg of a total lysate of gastric cells. Cleavage was detected using antibodies against the N-terminal extracellular and C-terminal cytoplasmic regions of JAM-A. The same protocol was followed for incubation of *H. pylori* sonicates with lysates of gastric cells. For MALDI-TOF/TOF, the protease activity of the selected fractions was tested against a synthetic 40-amino-acid JAM-A peptide (AYSRGHFDRTKKGTSSKKVIYSQPSARSEGEFKQTSSFLV, Genscript), corresponding to the full cytoplasmic domain of JAM-A. To characterize the cleavage, 1 µL of the peptide (0.15 µg/µL stock) was incubated with 9 µL of the chromatography fractions, for 15 minutes at 37°C. From this reaction, 2 µL of the digested sample was mixed with 2 µL of a CHCA matrix (8 mg/mL 50%ACN, 0.1% TFA). Then, 1 µL was spotted on the MALDI plate to detect peptide cleavage. Additional peptides were used for determining the cleavage site and site specificity (_277_KVIYSQPSARSEGEFK_292, 277_KVIYSQPSAKSEGEFK_292_, and _277_KVIYSQPSAGSEGEFK_292_, Genscript).

### Protease inhibition experiments

Inhibitors of different classes of proteases – namely, 1 µM E-64, 100 µM Leupeptin, 100 µM N-Tosyl-L-phenylalanine chloromethyl ketone (TPCK), 100 µM N-Tosyl-L-lysine chloromethyl ketone hydrochloride (TLCK), 0.3 µM Aprotinin, 1 µM Pepstatin A, 1 mM Phenylmethylsulfonyl fluoride (PMSF), 10 mM 1,10-Phenanthroline, and 5 mM Ethylenediamine tetraacetic acid (EDTA) – were used for a general characterization of the class of the proteases that cleaved JAM-A. All inhibitors were diluted in water, as methanol and DMSO inhibit the proteolytic effect. Twenty micrograms of *H. pylori* 26695 total lysate were incubated with 1 µg of the JAM-A peptide _277_KVIYSQPSARSEGEFK_292_, in the presence of each of the above protease inhibitors, for 15 minutes at 37°C. Digestion of the peptide was detected by MALDI-TOF analysis. The peptide alone and the peptide incubated with *H. pylori* without protease inhibitors were included as controls.

### Peptide Mass Fingerprint (PMF) and MS/MS

EC samples were 10x concentrated in an Amicon Ultra 0.5 mL, 10000 MWCO (Millipore) and further separated in a 10% SDS-PAGE. The gel was stained with Coomassie Blue. The protein bands were excised from the gel and processed for mass spectrometry analysis, as previously described.^51,52^ Briefly, bands were washed first with ultrapure water, then with 50% acetonitrile in 50 mM NH_4_HCO_3_, and were afterward dehydrated in 100% acetonitrile. Subsequently, proteins were reduced and alkylated by incubation in 25 mM dithiothreitol in 50 mM NH_4_HCO_3_, for a period of 20 minutes at 56°C, followed by incubation with 55 mM iodoacetamide in 50 mM NH_4_HCO_3_, for another 20 minutes at room temperature in the dark, before repeating the washing and dehydration procedures. Proteins were in gel digested with trypsin for 3 hours at 37°C in the presence of 0.01% surfactant (ProteaseMAX™, Promega), and the resulting peptides were extracted with 2.5% trifluoroacetic acid (TFA) for a period of 15 min at 1400 rpm (Thermomixer, Eppendorf), dried in a vacuum centrifuge concentrator (SpeedVac, Thermo Scientific) and resuspended in 0.1% TFA. For protein identification by MALDI mass spectrometry, a 4800 Plus MALDI TOF/TOF Analyzer (SCIEX) was used. Reversed-phase C18 chromatography (ZipTips, Millipore) was used to purify protein digests, which were eluted in a matrix constituted by alpha-Cyano-4-hydroxycinnamic acid (CHCA), dissolved in 50% acetonitrile, 0.1% TFA, 6 mM ammonium phosphate at 8 mg ml-1, in the MALDI sample plate. Peptide mass spectra data was collected in reflector positive mode in the m/z 700–5000 mass range. Some of the most intense peptide peaks associated with HP1012 and HP0657 proteins were selected for MS/MS peptide sequencing. Proteins were identified by the combined Peptide Mass Fingerprint and MS/MS peptide sequencing (PMF+MS/MS) approaches with the Mascot software (v2.6.1, Matrix Science), using the UniProt protein sequence database for the taxonomic selection Helicobacter pylori 26695 (2018_01 release).

### Cloning of proteases

The expression vector pGEX-6P-2 (GE Healthcare) was engineered to co-express HP1012 and HP0657, each with a different tag. To the original vector backbone, a synthetic 109-nucleotide sequence containing a Tac promoter, a ribosome binding site, a sequence of six histidines, and a new SacI restriction site was added (TGACAATTAATCATCGGCTCGTATAATGTGTGGAATTGTGAGCGGATAACAATTTCACACAGGAAACAGTATTCATGGGCAGCAGCCATCACCATCATCACCACAGCCA, Sigma), followed by the original EagI restriction sequence. The 109 ssDNA sample was converted to dsDNA by PCR, double-digested with SacI and EagI, and introduced into pGEX-6P-2. (The new vector was named pGEX_His.) All vectors used for protein expression are listed in Supplementary Table S8. For cloning and expression, *HP1012* and *HP0657* genes were amplified from the genomic DNA of *H. pylori* 26695, and the PCR products and the plasmid were digested with *EcoRI* and *XhoI* or with *SacI* and *EagI* (New England Biolabs). All primers used for cloning and sequencing are listed in Supplementary Table S7. Ligation of the PCR products with the pGEX-His plasmid was performed following the instructions for the Quick Ligation™ Kit (New England Biolabs). *E. coli* strain BL21 (DE3) (NZYtech) was transformed and plated on a Luria Broth Agar medium supplemented with 100 µg/mL of ampicillin (NZYtech). Transformants were selected upon overnight incubation at 37°C. Plasmid extraction was performed using the NZYMiniprep kit (NZYtech) following the manufacturer’s instructions.

### Site-directed mutagenesis

Recombinant HP0657 with deletion of the region spanning Gly_285_ to Tyr_307_, which includes the R/Y pair, and recombinant HP1012 with deletion of His_78_ to His_82_, and with substitutions of His_78_> Gln, Glu_81_> Gln, His_82_> Leu were obtained using the primers listed in Supplementary Table S7. For site directed mutagenesis, we followed the instructions of the Q5® Site-Directed Mutagenesis Kit (New England Biolabs). All DNA sequences were confirmed by Sanger sequencing, using primers listed in Supplementary Table S7.

### Expression and purification of recombinant proteases

Bacteria were grown on Luria broth media supplemented with 100 µg/mL of ampicillin (NZYtech) until reaching an optical density of 0.3 at 600 nm. Then, 100 uM Isopropyl β-D-1-thiogalactopyranoside (IPTG) (NZYtech) was added to the culture and incubated at 27°C overnight at 200 rpm. Bacteria were collected by centrifugation at 15000xg, for 15 min at 4°C. Pellets were suspended in ice cold PBS, pH 7.4. Bacteria lysis was obtained by sonication with 9 cycles of 3 pulses, each for 1 minute at 90% power. Bacterial debris were then removed by centrifugation, the supernatant was recovered and filtered in a 0.2 µm pore filter. Bacterial lysates were incubated with 100 µL of Glutathione Sepharose 4B overnight at 4°C and purified following the protocol described for GST SpinTrap (GE Healthcare), with the slight modification that for elution, we used PBS, pH7.4 with 20 mM reduced glutathione, pH 8.0.

### Statistical Analyses

Evaluation of the normality of distributions was performed with the Kolmogorov-Smirnov test. Normally distributed data were evaluated using the two-sided unpaired Student’s *t* test for comparisons between two independent groups, or by one-way or two-way analysis of variance (ANOVA) with the post-hoc Tukey’s test for comparisons between three independent groups. For data that did not follow a normal distribution, the Kruskal-Wallis test was used. The Mann-Whitney test was used to evaluate the relationship between JAM-A expression and the density of *H. pylori* colonization. Statistics were computed with SPSS (version 25) and GraphPad Prism (version 6.01). *P* < .05 was considered as statistically significant.

## Supplementary Material

Supplemental Material
