# Supplementary material for: Helicobacter pylori PqqE is a new virulence factor that cleaves junctional adhesion molecule A and disrupts gastric epithelial integrity
Source: Gut Microbes. 2021 May 10;13(1):1921928. doi: 10.1080/19490976.2021.1921928 (PMC8115454; doi:10.1080/19490976.2021.1921928)
Supplement: Supplemental Material [file KGMI_A_1921928_SM6788.pdf]

SUPPLEMENTAL MATERIAL

Helicobacter pylori PqqE is a new virulence factor that cleaves junctional adhesion molecule A and disrupts gastric epithelial integrity

Miguel S. Marques, Ana C. Costa, Hugo Osório, Marta L. Pinto, Sandra Relvas, Mário Dinis-Ribeiro, Fátima Carneiro, Marina Leite, Ceu Figueiredo*

Supplementary Figures S1 to S9
Supplementary Tables S1 to S8

SUPPLEMENTARY FIGURES

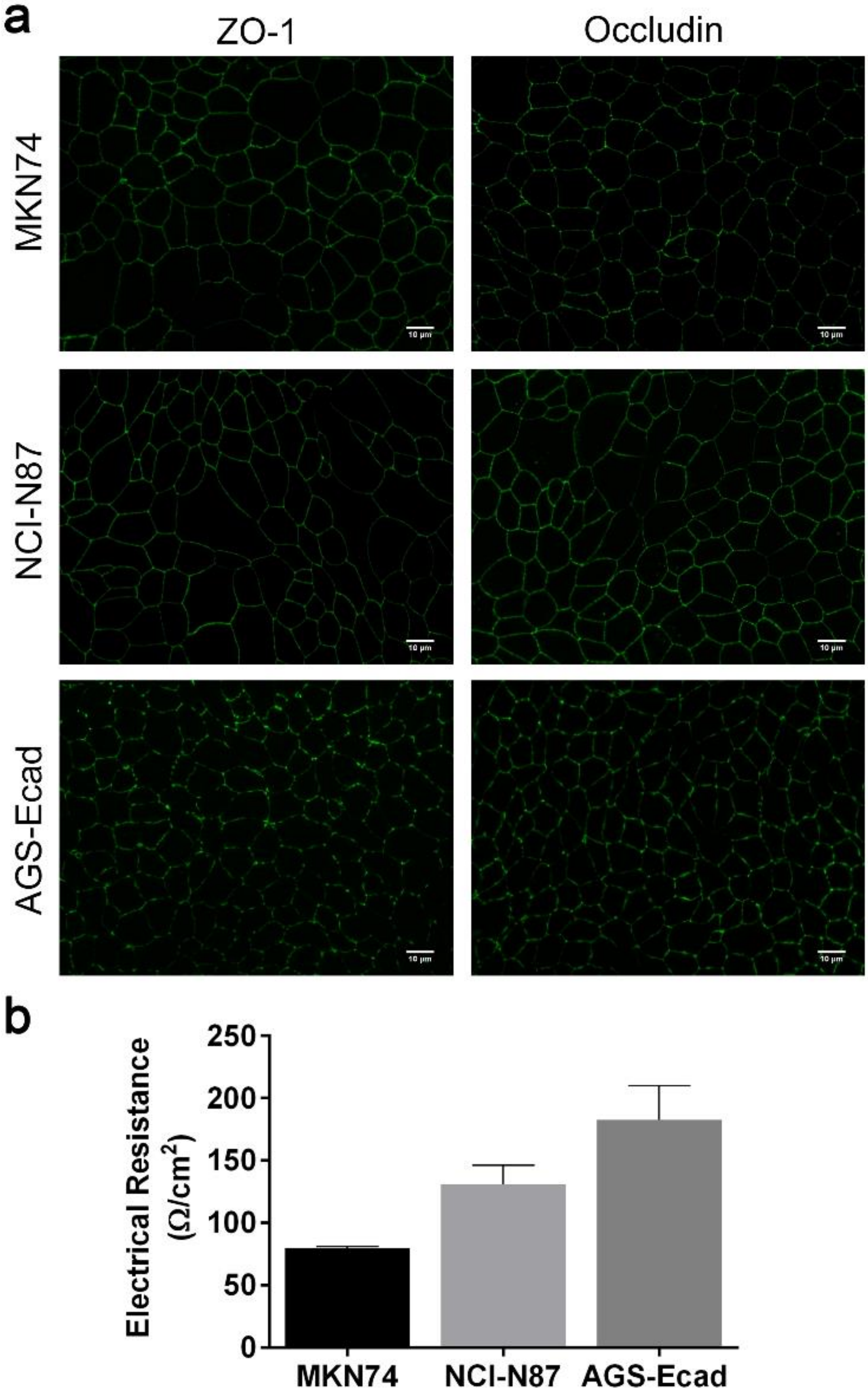

Supplementary Figure S1. MKN74, NCI-N87, and AGS-Ecad gastric cells establish competent tight junctions. a) Representative image of immunofluorescence of tight junction proteins ZO-1 and Occludin and **b)** transepithelial electrical resistance (TER) of monolayers after 6 days of confluence of the gastric cell lines MKN74, NCI-N87, and AGS-Ecad used in this study (n = 3). Data are represented by means ± s.e.m.

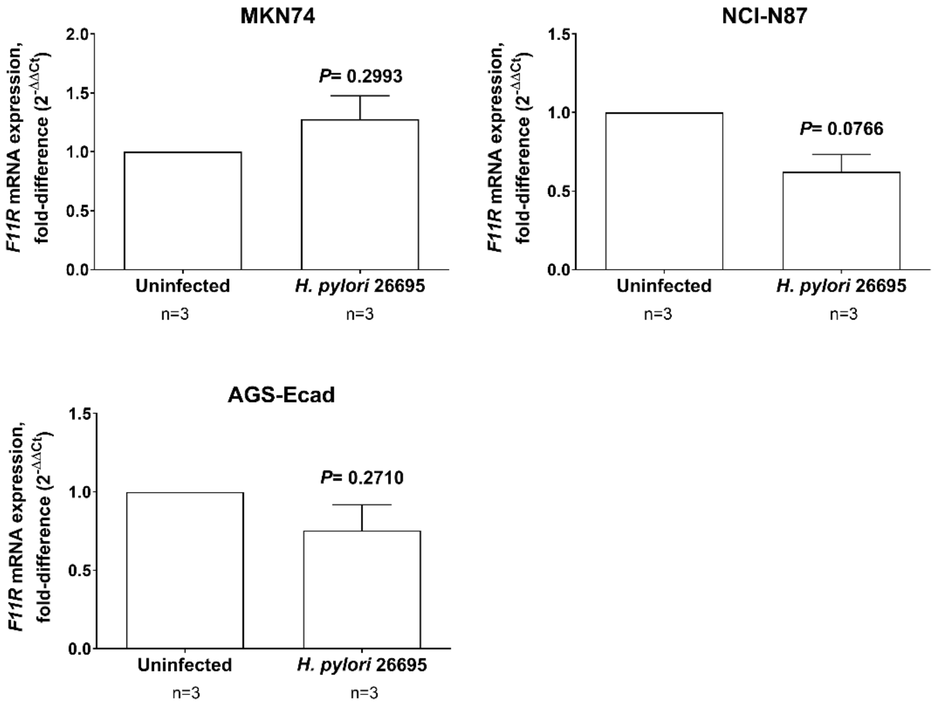

Supplementary Figure S2. *H. pylori* does not alter the mRNA expression of JAM-A in MKN74, NCI-N87, and AGS-Ecad gastric cell lines. MKN74, NCI-N87, and AGS-Ecad cells were infected with *H. pylori* 26695, and *F11R* expression was analyzed by real-time qPCR. *F11R* expression levels were normalized to GAPDH expression levels, and results are presented as the fold difference relative to uninfected MKN74, NCI-N87, or AGS-Ecad cells. Data correspond to mean values ± standard errors. Statistical significance was determined by the unpaired t test with Welch's correction, vs uninfected cells. Differences were considered statistically significant at P values of <0.05. Abbreviations: mRNA, messenger RNA; GAPDH, glyceraldehyde 3-phosphate dehydrogenase.

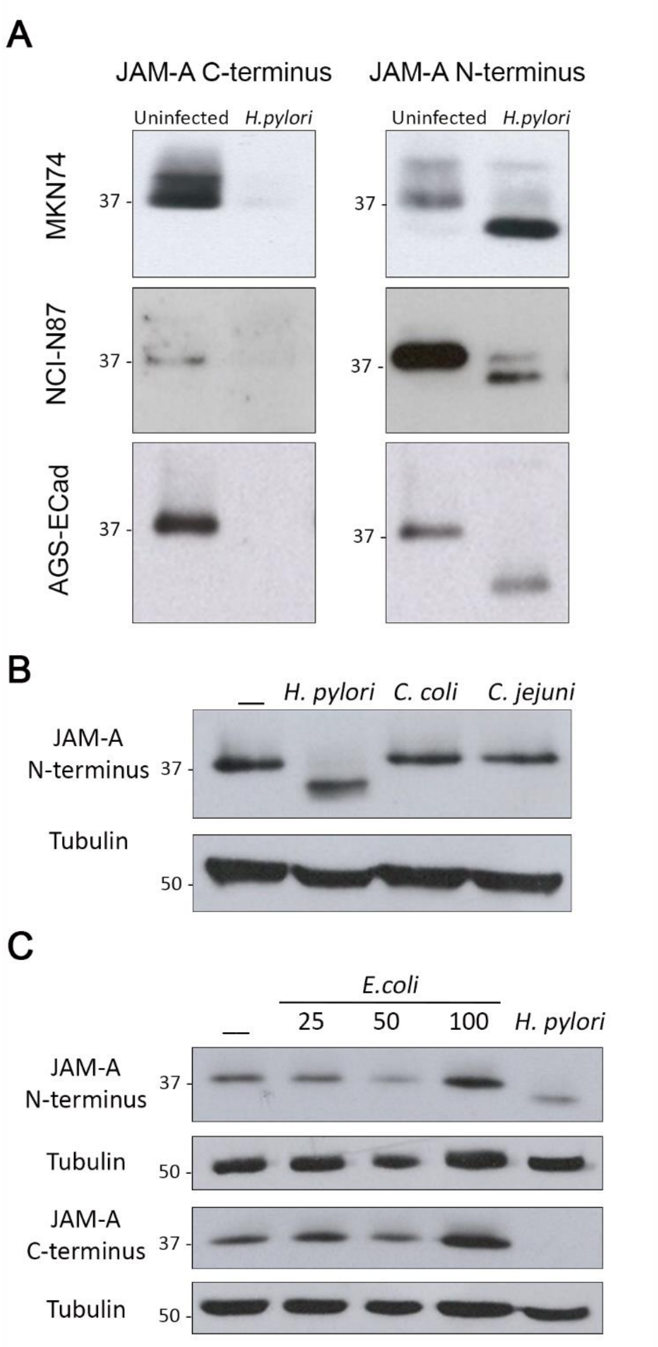

Supplementary Figure S3. Cleavage of the JAM-A cytoplasmic domain is independent of the gastric cell line used and is specific to *H. pylori*. a) Representative western blots of JAM-A using antibodies against the cytoplasmic C-terminus and the extracellular N-terminus domain of the protein, in total cell lysates of MKN74, NCI-N87, and AGS-Ecad gastric cells incubated (or not) with *H. pylori* 26695 lysates for 16 hours (6 hours in NCI-N87 cells). **b)** Western blot analysis of JAM-A in uninfected, *H. pylori* 26695-, *Campylobacter coli*-, or *C. jejuni*-infected AGS-Ecad cells using antibodies against the cytoplasmic C-terminus and the extracellular N-terminus domain of the protein. **c)** Western blot analysis of JAM-A in uninfected, *H. pylori* 26695-, or *Escherichia coli*-infected AGS-Ecad cells using antibodies against the cytoplasmic C-terminus and the extracellular N-terminus domain of the protein. *E. coli* was used at different MOIs (25, 50, and 100). *H. pylori* 26695 was used at a MOI of 100.

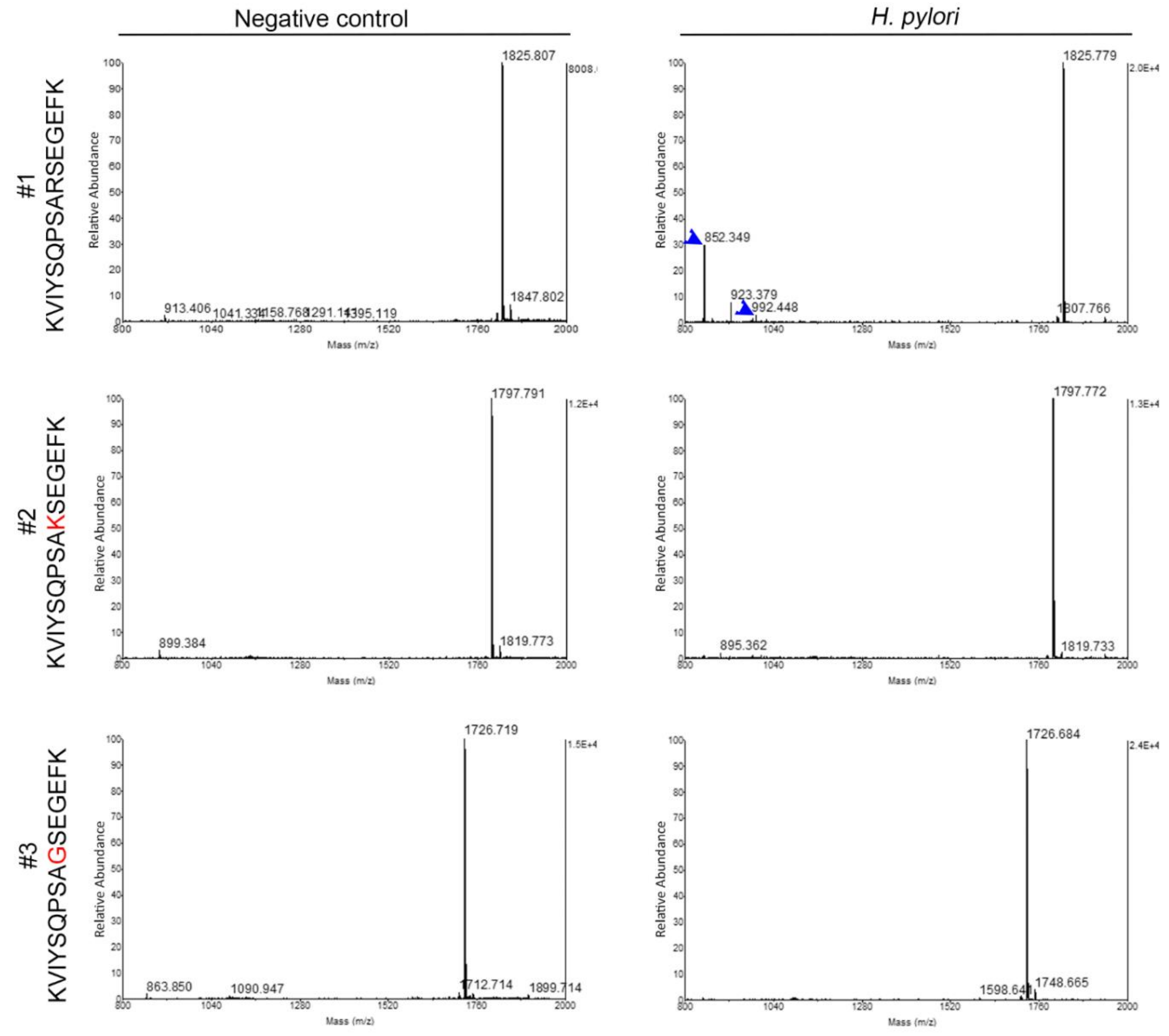

Supplementary Figure S4. Mass spectra obtained after incubation of *H. pylori* 26695 sonicates with three synthetic peptides corresponding to amino acids 277 to 292 of JAM-A. Peptide #1 is the wild-type control peptide ($_{277}$KVIYSQPSARSEGEFK$_{292}$), and peptides #2 and #3 contain substitutions in the amino acids at the cleavage site ($_{277}$KVIYSQPSA<u>K</u>SEGEFK$_{292}$ and $_{277}$KVIYSQPSA<u>G</u>SEGEFK$_{292}$). Incubations of the peptides with ultra-pure water were used for the negative controls. Peptides obtained from cleavage of the wild-type peptide are indicated by blue arrows, corresponding to *m/z* 852.3 and *m/z* 992.4.

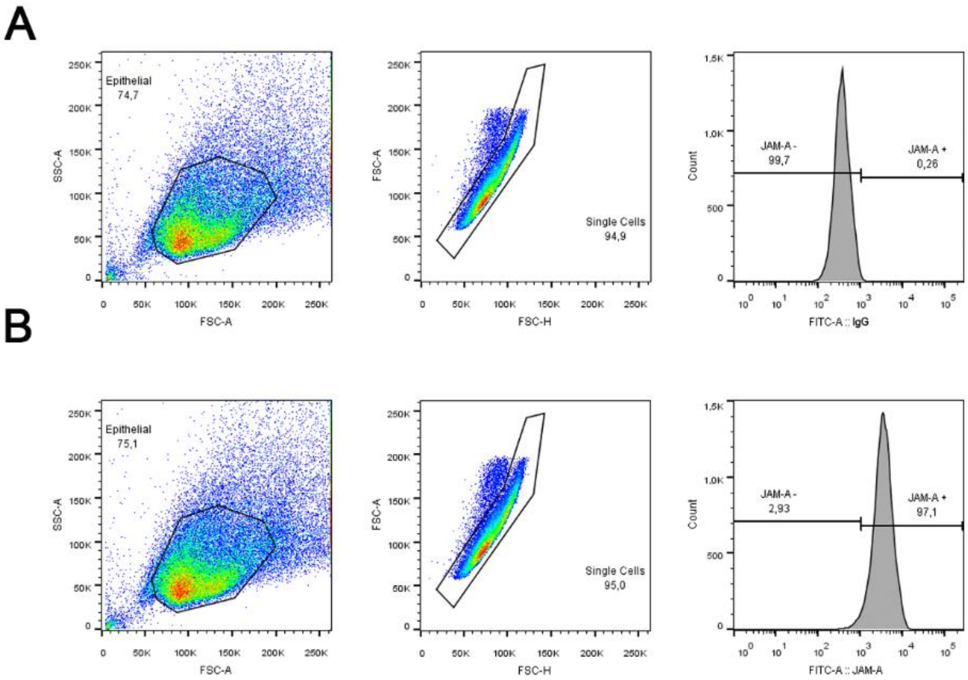

Supplementary Figure S5. Gating strategy used for flow cytometry analysis of JAM-A expression in CHO cells transfected with flJAM-A, sJAM-A, or Mock vectors. a-b) Samples stained with either anti-JAM-A (J10.4) FITC antibody or with IgG1 FITC isotype negative control antibody were analyzed by flow cytometry. Univariate histograms were generated upon double gating, first by forward scatter-Area (FSC-A) vs side scatter-Area (SSC-A) to exclude debris and dead cells from the cell population of interest, and second by FSC-Area vs FSC-Height (FSC-H) to select single cells and eliminate cell aggregates. A representative example of the gating strategy applied to CHO flJAM-A cells staining with the IgG1 FITC isotype antibody to define the negative population, and to CHO flJAM-A cells stained with anti-JAM-A (J10.4) FITC antibody to determine the JAM-A expression is depicted in **a)** and **b),** respectively.

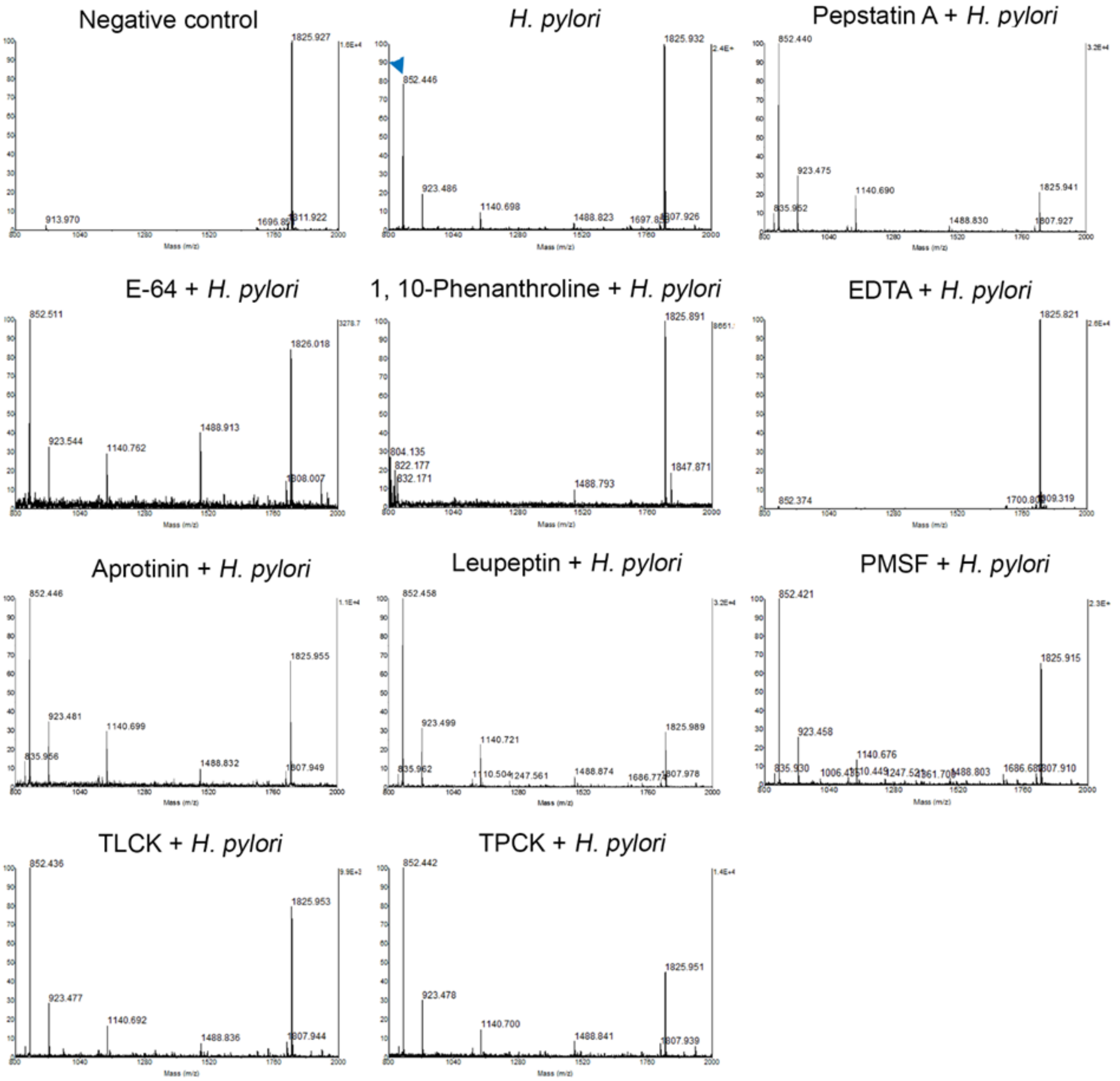

Supplementary Figure S6. Mass spectra obtained in the screening of the type of protease involved in *H. pylori*-mediated JAM-A cleavage. Incubations of *H. pylori* 26695 sonicates with the wild-type peptide ($_{277}$KVIYSQPSARSEGEFK$_{292}$) were performed in the presence of different protease inhibitors (Pepstatin A, E-64, 1,10-Phenanthroline, EDTA, Aprotinin, Leupeptin, PMSF, TLCK, or TPCK). Incubations of the peptide without protease inhibitors with *H. pylori* 26695 and with ultra-pure water were used as positive and negative controls, respectively. The blue arrow indicates the expected peak at *m/z* 852.4 obtained upon *H. pylori* cleavage. Due to short time of incubation, the peak at m/z 992.4 was not observed. yy' axis represents relative abundance. Experiments were performed at least twice, with similar results.

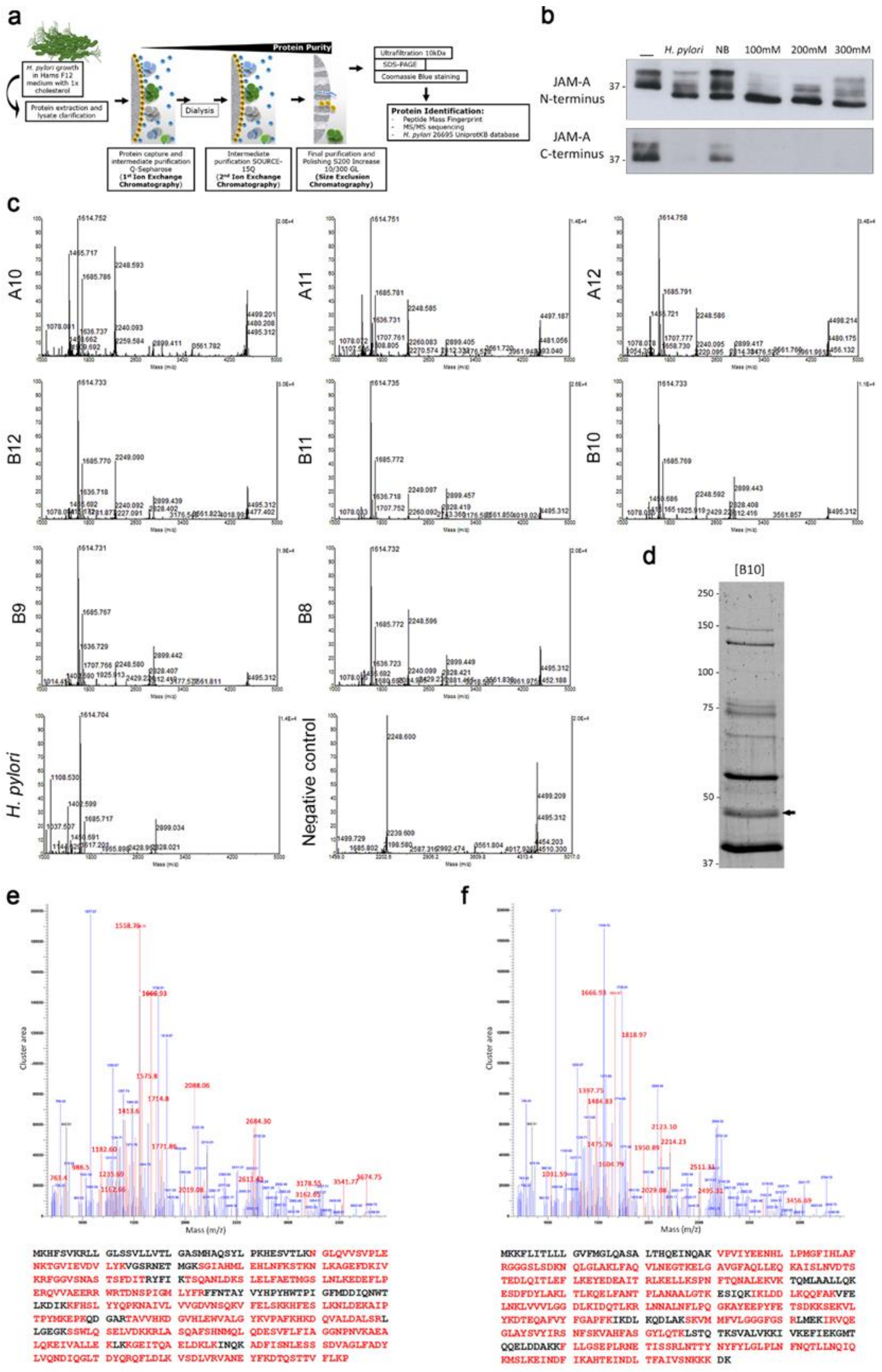

Supplementary Figure S7. Identification of the bacterial protease that cleaves the JAM-A cytoplasmic domain. a) Schematic representation of the protocol for purification and identification of the *H. pylori* protease that cleaves JAM-A, which involves two consecutive ion exchange

chromatographies, the first one using Q-sepharose and the second using Source-15Q, followed by size exclusion chromatography. After ultrafiltration, proteins were separated by SDS-PAGE and stained with Coomassie Blue. Protein bands are finally excised and identified by MALDI MS. **b)** After Q-sepharose purification, the non-bound (NB), and the 100mM, 200mM, and 300mM NaCl fractions were tested using MKN74 cell lysates, and cleavage of JAM-A monitored by western blot. *H. pylori* 25595 lysate was used as a control for cleavage of the JAM-A cytoplasmic domain. **c)** Mass spectra obtained after testing size exclusion chromatography (SEC) fractions (A10, A11, A12, B8, B9, B10, B11, and B12) for the presence of the bacterial protease, using the 40 amino acid peptide of the full cytoplasmic domain of JAM-A as substrate. yy' axis represents relative abundance. **d)** SDS-PAGE gel stained with Coomassie blue of the 10x concentrated B10 fraction obtained from SEC. The protein band indicated with an arrow was identified by mass spectrometry (PMF combined with peptide MS/MS sequencing). **e-f)** Mass spectra obtained from *in gel* tryptic digestion of the protein band resolved from B10 SEC fraction, which identified PqqE (HP1012) and YmxG (HP0657). **e)** Red peaks in the mass spectrum represent specific PqqE peptides; matched peptides in the PqqE sequence are shown in red, corresponding to 77% protein coverage data, obtained after PMF + MS/MS. **f)** Red peaks in the mass spectrum represent specific YmxG peptides; matched peptides in the YmxG are shown in red, corresponding to 76% protein coverage, obtained after PMF + MS/MS. Details on the peptide sequence and mass obtained for each protein can be found in Supplementary **Tables S4** and **S5**.

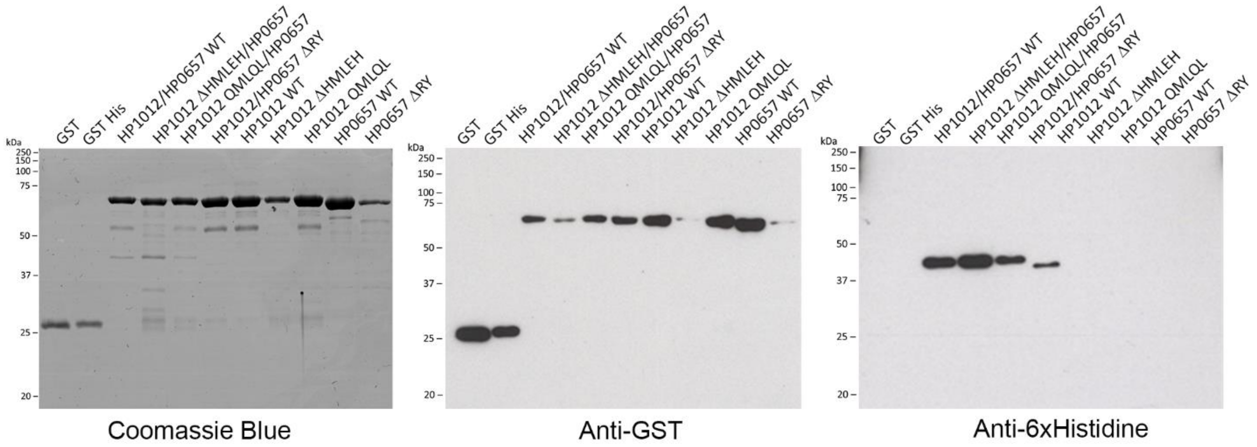

Supplementary Figure S8. Coomassie blue stained SDS-PAGE gel resolving the set of all recombinant bacterial proteases, expressed in the pGEX_His vector and purified by affinity chromatography (left). Western blot of the purified recombinant (single or combined) proteases using antibodies anti-GST (middle) and anti-6xHistidine (right).

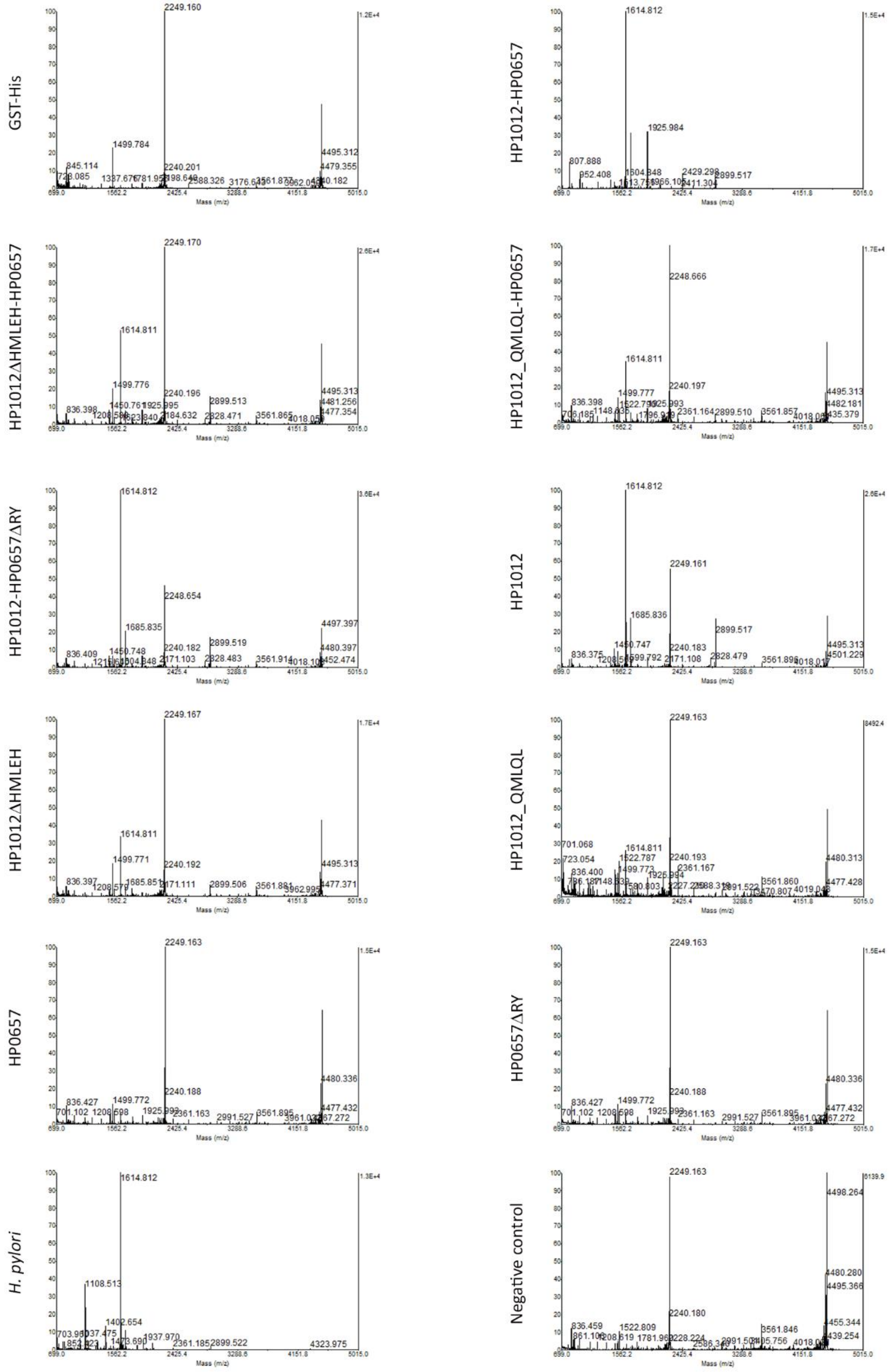

Supplementary Figure S9. Mass spectra obtained after incubation of the different recombinant proteases with the 40 amino acid peptide of the full cytoplasmic domain of JAM-A. Samples were incubated for 15 minutes at 37°C. yy’ axis represents relative abundance.

SUPPLEMENTARY TABLES

Supplementary Table S1. JAM-A (Q9Y624) coverage upon Peptide Mass Fingerprint analysis in uninfected conditions (Mascot score: 72; Expectation value: 0.0014, Matches: 11).

**Start – End**	**Observed mass (*m/z*)**	**ppm**	**Peptide**
60 – 71	1481.7035	1.05	R.VEWKFDQGDTTR.L
64 – 71	939.4153	-1.42	K.FDQGDTTR.L
79 – 86	954.4473	-5.61	K.ITASYEDR.V
79 – 97	2159.1487	4.82	K.ITASYEDRVTFLPTGITFK.S
87 – 97	1223.6952	-6.75	R.VTFLPTGITFK.S
126 – 148	2373.3743	-5.10	K.LIVLVPPSKPTVNIPSSATIGNR.A
216 – 228	1367.6356	-1.19	R.NGYGTPMTSNAVR.M
216 – 228	1383.6353	2.29	R.NGYGTPMTSNAVR.M + Oxidation (M)
277 – 286	1148.6421	-0.09	K.KVIYSQPSAR.S
278 – 286	1020.5479	0.63	K.VIYSQPSAR.S
287 – 299	1458.7065	-3.16	R.SEGEFKQTSSFLV.-

Supplementary Table S2. JAM-A (Q9Y624) coverage upon Peptide Mass Fingerprint analysis in *H. pylori* 26695 infected conditions (Mascot score: 56, Expectation value: 0.05, Matches: 8).

**Start – End**	**Observed mass (*m/z*)**	**ppm**	**Peptide**
60 – 71	1481.7084	4.36	R.VEWKFDQGDTTR.L
64 – 71	939.4216	5.29	K.FDQGDTTR.L
79 – 86	954.4567	4.25	K.ITASYEDR.V
79 – 97	2159.1167	-10.01	K.ITASYEDRVTFLPTGITFK.S
87 – 97	1223.6958	-6.26	R.VTFLPTGITFK.S
126 – 148	2373.3931	2.83	K.LIVLVPPSKPTVNIPSSATIGNR.A
216 – 228	1367.6323	-3.60	R.NGYGTPMTSNAVR.M
216 – 228	1383.6196	-9.07	R.NGYGTPMTSNAVR.M + Oxidation (M)

Supplementary Table S3. Raw data with the MS peak intensities of the detected ions of JAM-A (Q9Y624) in uninfected and *H. pylori* 26695 infected conditions. Cells in bold represent the 11 and the 8 JAM-A peaks observed upon peptide mass fingerprint analysis in each of the conditions and presented in Supplementary **Tables S1** and **S2.**

**Uninfected**	
m/z	**ion abundance**
720.4682	3085.5557
723.49353	2740.1997
724.47388	2863.709
726.41803	4984.7744
733.44763	3512.2563
734.43091	3725.2104
735.44977	2218.3743
736.41302	3188.6396
749.43427	3035.3025
750.54413	2313.3015
757.42371	4359.2227
759.44983	2365.9912
762.45673	2819.9263
763.46729	4762.4751
771.46173	3297.7383
776.53693	1953.9663
777.47174	2079.0452
779.45123	4096.9312
788.48047	3153.7256
802.45673	3351.9277
807.46136	3123.5603
825.09717	7650.6753
828.50989	1681.8604
832.31653	3419.8857
834.46606	3357.0647
842.5105	24228.363
849.49371	1708.1545
850.05933	1354.5464
854.0354	2120.2009
856.52795	3303.7515
861.05688	1837.288
864.50378	1543.5931
870.53973	15212.438
876.04413	1978.6882
877.03143	4262.4688
886.98822	1278.4764
907.24902	3616.2402
908.24573	931.56268
910.52429	1862.7478
**939.41534**	6322.7217
950.52582	1867.6459
**954.44733**	1206.0217
977.48389	1174.5363
994.13849	1279.8145
**1020.5479**	14633.621
1045.5662	3668.9355
1064.592	1633.599
1099.4611	2255.0669
1101.6595	1753.5026
**1148.6421**	21429.756
**1223.6952**	2457.8733
1243.6616	7924.5117
1265.6477	2280.3372
1274.5826	1685.3083
1301.7338	5574.8901
1321.8259	3562.4392
1322.8169	8060.4814
1326.6639	12961.394
1345.6228	1435.3439
**1367.6356**	2835.3379
1368.6251	3735.3594
**1383.6353**	4032.9607
1384.6206	3954.6135
**1458.7065**	4858.6152
1480.7003	2824.1936
**1481.7035**	1666.4539
1496.7465	925.4201
1497.6926	4293.6001
1527.7947	1271.2644
1566.7483	1470.0199
1573.7888	1604.9335
1766.8256	1143.3347
1797.9508	1547.1434
1813.9587	2425.9958

***H. pylori*-infected**	
m/z	**ion abundance**
741.41968	1827.4277
745.38379	1471.3339
804.30487	2611.5337
832.33441	5457.9443
833.3313	1431.1858
842.50848	34067.496
856.5296	3124.6631
864.4859	1840.9149
870.53943	19782.418
927.49573	1976.771
**939.42163**	3629.9136
**954.45673**	1790.8903
1045.5608	6078.5586
1047.5878	918.20581
1099.4673	5594.8149
1149.5924	732.7937
**1223.6958**	920.12189
1243.6664	3058.8931
1301.7281	5411.7959
1307.694	1285.7758
1321.8344	2406.2002
1322.8246	2571.7808
1326.6635	6983.2808
1337.5889	3269.6665
1345.6228	2036.9989
1350.6017	1474.7915
**1367.6323**	3000.9907
1368.625	2865.6113
**1383.6196**	2661.2937
1385.6067	800.4364
1426.7598	892.15881
1479.791	1328.4041
1480.7864	1688.1682
**1481.7084**	4247.8384
1557.8253	549.77319
1573.7826	849.91296
1574.7881	732.51324
1605.8308	1818.0408
1639.9246	1682.0447
1731.8673	1669.8066
1765.8171	786.58936
1797.9468	1068.871
1813.9611	1562.8965
1819.9225	989.41821
1854.9712	719.97333
1940.9489	904.32172
1965.9111	631.98065
1982.9149	1026.3143
2107.981	551.96869
**2159.1167**	1032.0651
2208.1628	1623.9285
2211.1433	931.14392
2212.1213	642.83124
2218.1702	2268.3831
2233.1143	778.5
**2373.3931**	1569.6642
2381.0066	495.52518
2437.2393	526.19269
2438.3853	1199.8235
2574.9507	551.91669
2603.0645	590.6394
2607.5317	201.57787
2684.0217	185.71817
2729.1753	290.7771

1820.9592	762.86023
1939.9293	537.26782
1940.955	916.53162
1965.894	1200.9309
1981.907	2361.293
**2159.1487**	802.94794
2208.1897	1068.5553
2211.1252	1464.7001
2218.145	2607.467
2319.8193	475.5419
**2373.3743**	2622.7598
2437.2957	2459.1592
2602.0591	2678.9192
2764.1587	1553.7443
3271.3726	262.38092
3786.8582	222.23474
3913.9375	266.81677

Supplementary Table S4. Peptides obtained for O25656; Protease PqqE (HP1012) OS=*Helicobacter pylori* (strain ATCC700392 / 26695) upon PMF+MS/MS analysis.

Start – End	Observed mass (*m/z*)	Peptide	MS/MS ion score
40 – 52	1396.7781	K.NGLQVVSVPLENK.T	
40 – 63	2613.4346	K.NGLQVVSVPLENKTGVIEVDVLYK.V	
53 – 63	1235.6877	K.TGVIEVDVLYK.V	
74 – 86	1496.7700	K.SGIAHMLEHLNFK.S	13
74 – 86	1512.7640	K.SGIAHMLEHLNFK.S + Oxidation (M)	16
87 – 98	1337.7168	K.STKNLKAGEFDK.I	
93 – 102	1162.6631	K.AGEFDKIVKR.F	
102 – 117	1714.8510	K.RFGGVSNASTSFDITR.Y	
103 – 117	1558.7540	R.FGGVSNASTSFDITR.Y	32
122 – 144	2510.3057	K.TSQANLDKSLELFAETMGSLNLK.E	
122 – 152	3525.7168	K.TSQANLDKSLELFAETMGSLNLKEDEFLPER.Q	
122 – 152	3541.7660	K.TSQANLDKSLELFAETMGSLNLKEDEFLPER.Q + Oxidation (M)	
130 – 152	2668.3020	K.SLELFAETMGSLNLKEDEFLPER.Q	16
130 – 152	2684.2980	K.SLELFAETMGSLNLKEDEFLPER.Q + Oxidation (M)	35
130 – 159	3495.6936	K.SLELFAETMGSLNLKEDEFLPERQVVAEER.R + Oxidation (M)	
153 – 159	830.4414	R.QVVAEER.R	
153 – 160	986.5381	R.QVVAEERR.W	
153 – 162	1328.7327	R.QVVAEERRWR.T	
161 – 174	1755.8640	R.WRTDNSPIGMLYFR.F	
161 – 174	1771.8573	R.WRTDNSPIGMLYFR.F + Oxidation (M)	
163 – 174	1413.6891	R.TDNSPIGMLYFR.F	
163 – 174	1429.6800	R.TDNSPIGMLYFR.F + Oxidation (M)	0
206 – 215	1310.6904	K.KFHSLYYQPK.N	
207 – 215	1182.5970	K.FHSLYYQPK.N	9
216 – 229	1455.8175	K.NAIVLVVGDVNSQK.V	
216 – 235	2159.2004	K.NAIVLVVGDVNSQKVFELSK.K	
236 – 247	1487.8008	K.KHFESLKNLDEK.A	
248 – 258	1274.6793	K.AIPTPYMKEPK.Q	
248 – 258	1290.6776	K.AIPTPYMKEPK.Q + Oxidation (M)	
264 – 282	2122.1370	R.TAVVHKDGVHLEWVALGYK.V	
270 – 287	2029.0835	K.DGVHLEWVALGYKVPAFK.H	
288 – 299	1352.7330	K.HKDQVALDALSR.L	22
306 – 316	1291.6672	K.SSWLQSELVDK.K	
306 – 318	1575.8500	K.SSWLQSELVDKKR.L	16
319 – 347	3162.5505	R.LASQAFSHNMQLQDESVFLFIAGGNPNVK.A	
319 – 347	3178.5510	R.LASQAFSHNMQLQDESVFLFIAGGNPNVK.A + Oxidation (M)	
348 – 361	1554.9028	K.AEALQKEIVALLEK.L	
364 – 376	1472.8221	K.KGEITQAELDKLK.I	
381 – 414	3764.7510	K.ADFISNLESSSDVAGLFADYLVQNDIQGLTDYQR.Q	23
415 – 420	763.4340	R.QFLDLK.V	
415 – 426	1432.8167	R.QFLDLKVSDLVR.V	
427 – 444	2088.0570	R.VANEYFKDTQSTTVFLKP.-	53

Supplementary Table S5. Peptides obtained for O25371; Processing protease YmxG (HP0657) OS=*Helicobacter pylori* (strain ATCC700392 / 26695) upon PMF+MS/MS analysis.

Start – End	Observed mass (*m/z*)	Peptide	MS/MS ion score
31 – 51	2495.3147	K.VPVIYEENHLLPMGFIHLAFR.G	
31 – 51	2511.3093	K.VPVIYEENHLLPMGFIHLAFR.G + Oxidation (M)	
52 – 66	1444.7759	R.GGGSLSDKNQLGLAK.L	
67 – 77	1219.6687	K.LFAQVLNEGTK.E	
67 – 91	2703.4490	K.LFAQVLNEGTKELGAVGFAQLLEQK.A	
78 – 91	1502.8191	K.ELGAVGFAQLLEQK.A	
92 – 112	2350.2222	K.AISLNVDTSTEDLQITLEFLK.E	
92 – 121	3456.6919	K.AISLNVDTSTEDLQITLEFLKEYEDEAITR.L	
122 – 138	1973.0966	R.LKELLKSPNFTQNALEK.V	
128 – 140	1475.7632	K.SPNFTQNALEKVK.T	
151 – 159	1087.5020	K.ESDFDYLAK.L	
160 – 180	2214.2340	K.LTLKQELFANTPLANAALGTK.E	0
186 – 197	1446.8295	K.IKLDDLKQQFAK.V	
204 – 220	1867.1376	K.LVVVLGGDLKIDQTLKR.L	
220 – 232	1484.8340	K.RLNNALNFLPQGK.A	6
221 – 232	1328.7327	R.LNNALNFLPQGK.A	
233 – 244	1478.6376	K.AYEEPYFETSDK.K	
233 – 248	1950.8940	K.AYEEPYFETSDKKSEK.V	18
249 – 266	2123.0990	K.VLYKDTEQAFVYFGAPFK.I	27
253 – 266	1619.7759	K.DTEQAFVYFGAPFK.I	
277 – 291	1588.7974	K.SKVMMFVLGGGFGSR.L + Oxidation (M)	
277 – 291	1604.7925	K.SKVMMFVLGGGFGSR.L + 2 Oxidation (M)	
279 – 291	1357.6937	K.VMMFVLGGGFGSR.L	
279 – 291	1373.6719	K.VMMFVLGGGFGSR.L + Oxidation (M)	
279 – 291	1389.6658	K.VMMFVLGGGFGSR.L + 2 Oxidation (M)	
296 – 309	1666.9291	K.IRVQEGLAYSVYIR.S	
298 – 309	1397.7510	R.VQEGLAYSVYIR.S	15
310 – 326	1884.9551	R.SNFSKVAHFASGYLQTK.L	
315 – 326	1321.6921	K.VAHFASGYLQTK.L	
360 – 368	1031.5925	K.FLLGSEPLR.N	
360 – 375	1818.9660	K.FLLGSEPLRNETISSR.L	32
376 – 401	3133.6104	R.LNTTYNYFYLGLPLNFNQTLLNQIQK.M	
402 – 412	1337.7168	K.MSLKEINDFIK.A	
413 – 430	2029.0835	K.AHTEINDLTFAIVSNKKK.D	

Supplementary Table S6. Predicted functional partners of PqqE (HP1012) from *H. pylori* 26695 using the STRING database.

**Predicted Functional Partners of PqqE**		**Score**
HP_0657	Processing protease (ymxG)	0.997
fbcH	Ubiquinol cytochrome c oxidoreductase, cytochrome c1	0.991
HP_1540	Ubiquinol cytochrome c oxidoreductase, Rieske 2Fe-2S subunit (fbcF)	0.991
HP_1539	Ubiquinol cytochrome c oxidoreductase, cytochrome b subunit (fbcH)	0.963
atpD	ATP synthase F0F1 subunit beta	0.963
atpA	ATP synthase F0F1 subunit alpha	0.914
pyrD	Dihydroorotate dehydrogenase 2	0.906
HP_1014	7-alpha-hydroxysteroid dehydrogenase	0.893
dapA	Dihydrodipicolinate synthase	0.893
HP_1135	ATP synthase F0F1 subunit delta	0.890

Supplementary Table S7. List of primers used in this study. Restriction enzymes location are underlined.

Primer Name	Sequence	Function
**Cloning**		
PqqE_FñSP_EcoRI	TCGAGAATTCGCATGCACGCACAATCTTACT	HP1012 without signal peptide
R1PqqE_XhoI	AGCTCTCGAGGGCGTAATCAACGCGCTAG	
F1_HP0657_SacI	GGCCGAGCTCTCCGGCTCAAAGCAAAGGTA	HP0657 without signal peptide
R1_HP0657_EagI	GGCCCGGCCGATTACAGCTTCAAATGGCATCA	
F_HP0657_EcoRI	TCGAGAATTCCTTTGACACACCAAGAAATCAA	HP0657 without signal peptide
R_HP0657_XhoI	AGCTCTCGAGATTACAGCTTCAAATGGCATCA	
**Sequencing**		
pGEX-5’	GGGCTGGCAAGCCACGTTTGGTG	pGEX-6P-2
pGEX-3’	CCGGGAGCTGCATGTGTCAGAGG	
SR2_PqqE	CTTCAGCGACCACTTGACGCT	HP1012
SF3_PqqE	AACCAGTCAGGCTAACTTGGAT	
SR3_PqqE	CACGCTTTCATCTTGTAATTGCA	
SF4_PqqE	GCGAAGGCAAAAGCTCGTGG	
Rs1_HP0657	TTAGCCAAATAGTCAAAATCGC	HP0657
Fs2_HP0657	GTGGATACCAGCACAGAAGA	
Rs2_HP0657	TTTAGATTTCGCTAAATCCTGTT	
Fs3_HP0657	GTCCTCTATAAAGACACTGAG	
M13_Fw	GTAAAACGACGGCCAG	F11R
M13_Rv	CAGGAAACAGCTATGAC	
**Plasmid building blocks**		
F_XhoI_His.frag	TCGACTCGAGTGACAATTAATCATCGGCTCGT	pGEX_His_Frag
R_EagI/SacI_His.frag	GGCCGCGGCCGCGAGCTCTGGCTGTGGTGATGATGGTG	
**Site directed mutagenesis**		
Q5SDM_DelHMLEH_F	TTGAATTTTAAAAGCACCAAAAACCTTAAAG	Deletion of HP1012 HMLEH
JQ5SDM_ DelHMLEH _R	AGCGATCCCGCTCTTTCC	
Q5SDM_PqqE_F	ACAACTTTTGAATTTTAAAAGCACCAAAAACC	Substitution of HP1012 HMLEH>QMLQL
Q5SDM_PqqE_R	AACATCTGAGCGATCCCGCTCTTT	
Q5SDM_YmxG-Del_F	TATCCGCTCCAATTTTTCTAAAG	Deletion of HP0657 RY region
Q5SDM_YmxG-Del_R	AGCACAAACATCATGACTTTAG	

Supplementary Table S8. List of plasmids used in this study.

Plasmid name	Function
pENTR Directional TOPO (Invitrogen)	Cloning of flJAM-A and sJAM-A
pEF6/*Myc*-His (Invitrogen)	Expression of flJAM-A and sJAM-A
pGEX-6P-2 (GE Healthcare)	Cloning with GST tag
pGEX-His	Double cloning with GST or Histidine tag
pGEX_PqqE_His_YmxG	GST-HP1012 and His-HP0657 expression
pGEX_PqqE (ΔHMLEH)_His_YmxG	GST-HP1012 (ΔHMLEH) and His-HP0657 expression
pGEX_PqqE (QMLQL)_His_YmxG	GST-HP1012 (QMLQL) and His-HP0657 expression
pGEX_PqqE_His_YmxG (ΔRY)	GST-HP1012 and His-HP0657 (ΔRY)expression
pGEX_PqqE_His	GST-HP1012
pGEX_PqqE (ΔHMLEH)_His	GST-HP1012 (ΔHMLEH)
pGEX_PqqE (QMLQL)_His	GST-HP1012 (QMLQL)
pGEX_YmxG_His	GST-HP0657 expression
pGEX_YmxG (ΔRY)_His	GST-HP0657 (ΔRY) expression
